# Supplementary material for: ETMR stem-like state and chemo-resistance are supported by perivascular cells at single-cell resolution
Source: Nat Commun. 2025 Jun 25;16:5394. doi: 10.1038/s41467-025-60442-9 (PMC12198369; doi:10.1038/s41467-025-60442-9)
Supplement: Supplementary file 1 — Supplementary Information [file 41467_2025_60442_MOESM1_ESM.pdf]

SUPPLEMENTARY FIGURES

ETMR stem-like state and chemo-resistance are supported by perivascular cells at single-cell resolution

Flavia W. de Faria[1*], Nicole C. Riedel[1*], Daniel Münter[1], Marta Interlandi[1,2], Carolin Göbel[3,4], Lea Altendorf[3,4]; Mathis Richter[5], Viktoria Melcher[1], Christian Thomas[6], Rajanya Roy[1], Melanie Schoof[3,4], Ivan Bedzhov [7], Natalia Moreno[1], Monika Graf [1], Marc Hotfilder[1], Dörthe Holdhof [3,4], Wolfgang Hartmann[8, 9], Ann-Katrin Bruns[10], Angela Brentrup[10], Friederike Liesche-Starnecker[11], Bruno Maerkl[11], Sarah Sandmann[2], Julian Varghese[2], Martin Dugas[2,12], Pedro H. Pinto[13], Sebastian T. Balbach[1], I-Na Lu[1], Claudia Rossig[1], Oliver Soehnlein[5], Aysegül Canak[14], Martin Ebinger[15], Martin Schuhmann[15], Jens Schittenhelm[16], Michael F. Frühwald[17], Ulrich Schüller[3,4], Thomas K. Albert[1], Carolin Walter[1,2§], Jan M. Bruder[18§], Kornelius Kerl[1§]

*Correspondence: kornelius.kerl@ukmuenster.de

[1] Department of Pediatric Hematology and Oncology, University Hospital Münster, Münster, Germany. [2] Institute of Medical Informatics, Westphalian Wilhelms University Münster, Münster, Germany. [3] Department of Pediatric Hematology and Oncology, University Medical Center Hamburg-Eppendorf, Hamburg, Germany. [4] Research Institute Children's Cancer Center Hamburg, Hamburg, Germany. [5] Institute for Experimental Pathology, Center for Molecular Biology of Inflammation, University of Münster, Münster, Germany. [6] Institute of Neuropathology, University Hospital Münster, Münster, Germany. [7] Embryonic Self-Organization Research Group, Max Planck Institute for Molecular Biomedicine, Münster, Germany. [8] Division of Translational Pathology, Gerhard-Domagk Institute of Pathology, University Hospital Münster, Münster, Germany. [9] West German Cancer Center (WTZ), Network Partner Site, University Hospital Münster, Münster, Germany. [10] Department of Neurosurgery, University Hospital Münster, Münster, Germany. [11] Department of Neuropathology, Pathology, Medical Faculty, University of Augsburg, Augsburg, Germany. [12] Institute of Medical Informatics, Heidelberg University Hospital, Heidelberg, Germany. [13] Department of Pathology, Children's Hospital of Brasilia Jose de Alencar, Brasilia, Brazil. [14] Department of Hematology and Oncology, Children's University Hospital Tübingen, and German Cancer Consortium (DKTK) Tübingen, Tübingen, Germany. [15] Department of Neurosurgery, section of Pediatric Neurosurgery, University Hospital Tübingen, and German Cancer Consortium (DKTK) Tübingen, Tübingen, Germany. [16] Department of Neuropathology, University Medical Hospital, Eberhard Karls University Tübingen, Tübingen, Germany. [17] Swabian Children's Cancer Center, Pediatric and Adolescent Medicine, University Center Augsburg, Augsburg, Germany. [18] Department for Cell and Developmental Biology, Max Planck Institute for molecular Biomedicine, Münster, Germany. *These authos contributed equally: Flavia W. de Faria, Nicole C. Riedel. [§]These authors jointly supervised this work: Carolin Walter, Jan M Bruder, Kornelius Kerl. Corresponding author and lead contact: Kornelius Kerl

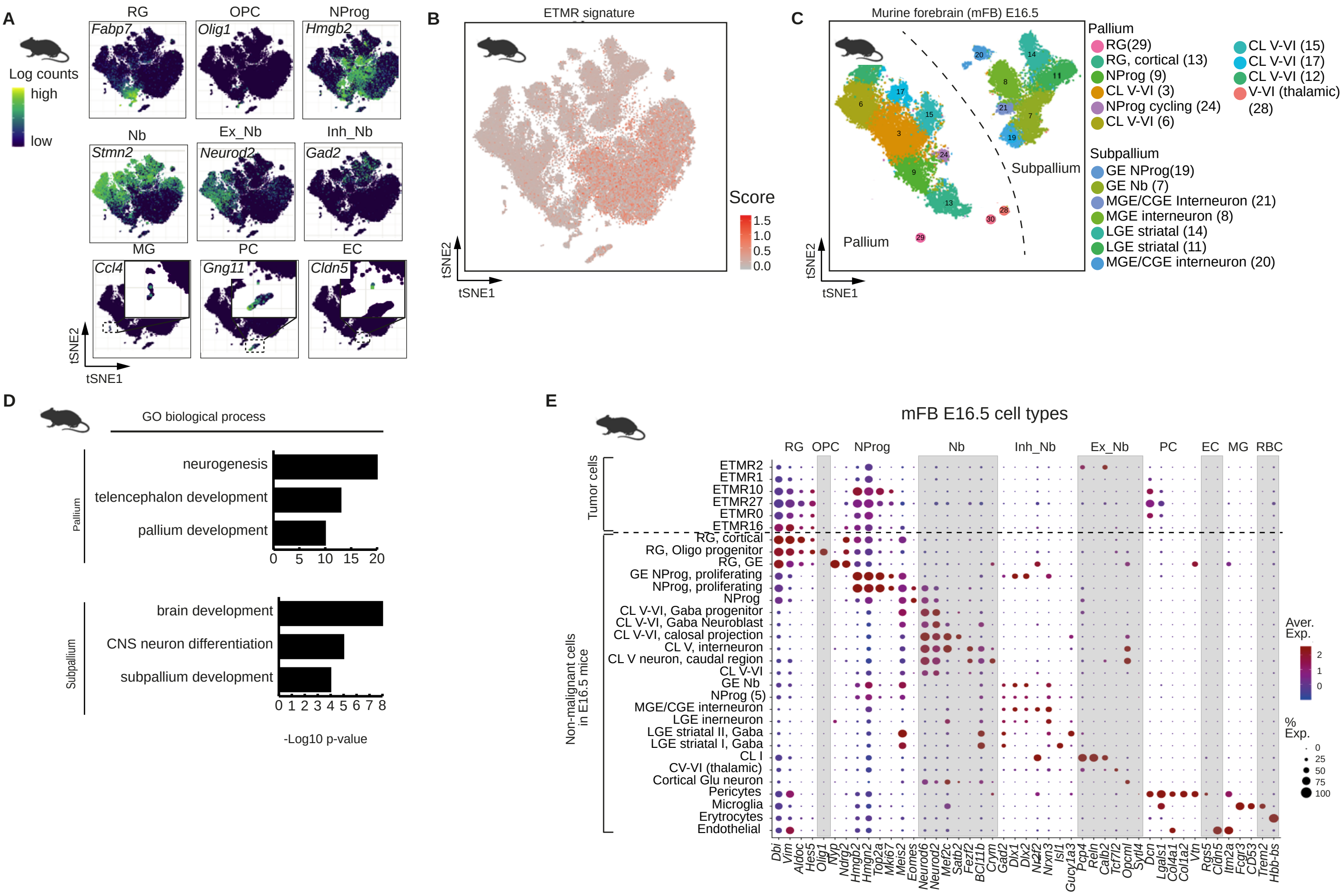

Supplementary Fig. 1. Murine E16.5 forebrain lineage diversity and ETMR cell annotation. **A**, t-SNE plots displaying the expression levels of representative marker genes of the main cell types found in the murine dataset (also refer to Figure 1C): RG (Fabp7), OPC (Olig1), NProg (Hmgb2), Nb (Stmn2), Ex_Neu (Neurod2), Inh_Neu (Gad2), MG (Ccl4), PC (Gng11), EC (Cldn5). Color-coded by enrichment score. **B**, t-SNE plot depicting the murine ETMR-signature of murine forebrain ETMR samples from Fig. 1C and Suppl. fig. S1A. Color-coded by gene enrichment score. **C**, t-SNE representation of the cells from murine forebrain samples which are cre-recombinase negative and serve as control samples not harboring ETMR. Cell annotation of the murine E16.5 forebrain with the descriptive cell subtypes, color-coded by unsupervised clustering. Dashed line delineates the pallium and subpallium clustering compartments. Also refer to figure 1B-C. **D**, Bar graph describing the most significant gene ontology (GO) terms from all upregulated genes related to the two distinct brain regions (pallium versus subpallium) from (C). **E**, Dot plot represents the marker genes for the identified cell types and their expression level in the integrated murine dataset (see Fig. 1B and 1C). Dots' size represents the percentage of cells within a cluster expressing the gene. Color intensity defines the average expression level of the indicated gene per cell type. Abbreviations: RG: radial glia cell; OPC: oligoprogenitor cell; NProg: neuroprogenitor; Nb: neuroblast; Ex_Neu: excitatory neurblast / neuron; Inh_Neu: inhibitory neuroblast / neuron; MG: microglia; PC: pericytes; EC: endothelial cell; CL V-VI: cortical layer V-VI; GE: gangli-on-ic eminence; GE NProg: neuroprogenitors derived from the GE; GE Nb: neuroblasts derived from the GE; MGE: medial GE; CGE: caudal GE; LGE: lateral GE. Source data of **D** is provided as a Source Data file. Sample type symbols in **A**-**E** created with Biorender.com

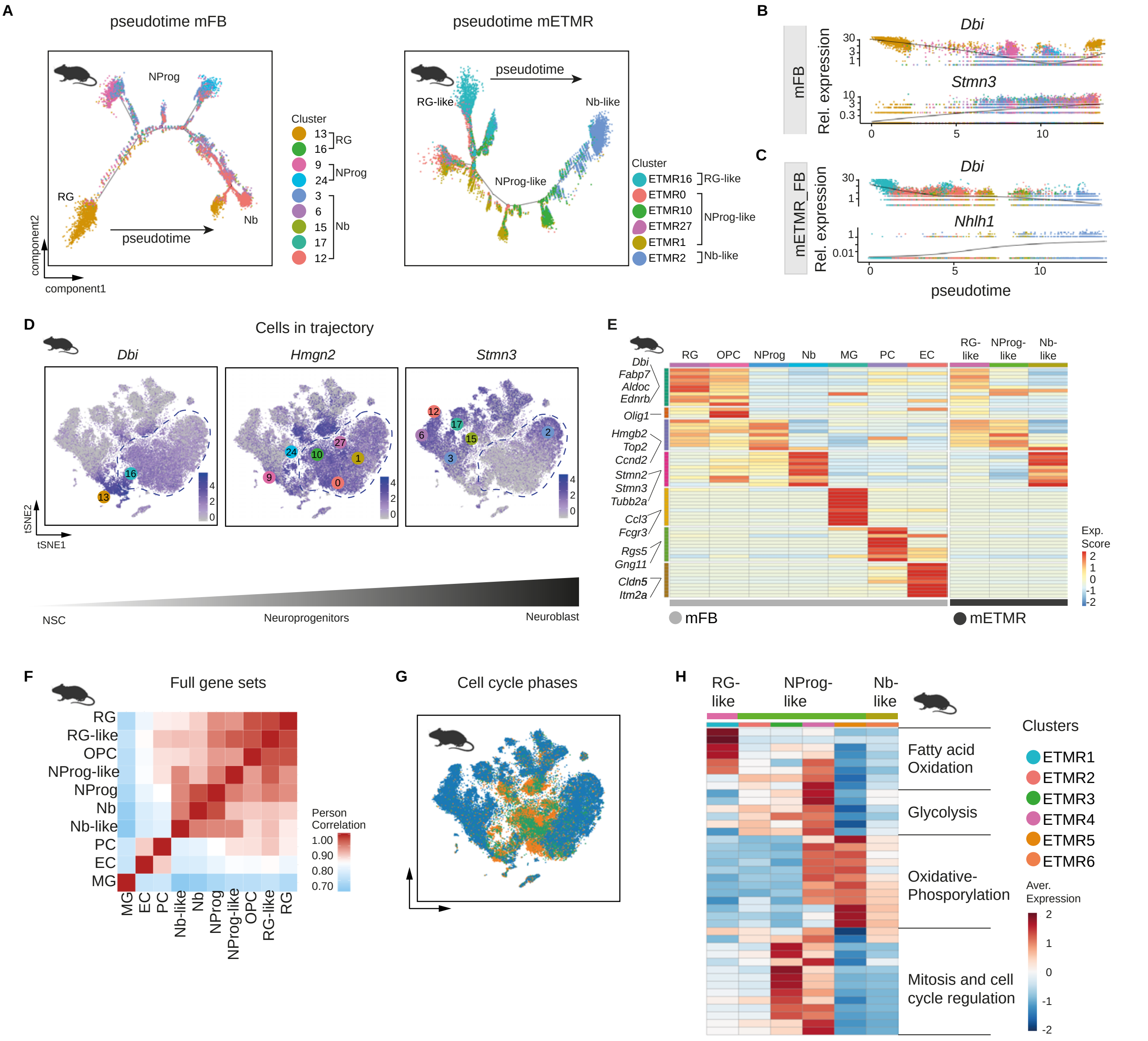

Supplementary Fig. 2. Murine ETMR cells followed a neurodevelopmental timeline during the genesis of their subpopulations.A, Pseudotime analysis of the wild-type murine control forebrain cells (left) and ETMR malignant cells (right). Color-coded by unsupervised clustering. Arrows indicate the direction of pseudotime in the trajectory. **B-C**, Graphs representing the relative expression of genes in the pseudotime trajectories of the mFB (B) and mETMR (C). Depicted the genes mostly enriched at the beginning or end of the trajectories. *Dbi* is a marker of RG cells; *Stmn3* and *Nhlh1* markers post-mitotic Nb. Color-coded as in (A). **D**, t-SNE showing the expression level of *Dbi* (RG marker), *Hmgn2* (NProg marker), and *Stmn3* (Nb marker). The genes follow the gradient of distinct neuronal develop-mental states: RG, NProg, and Nb. Blue dashed lines delineate ETMR malignant cellular populations. Numbers correspondent to clusters defined in (A). Color-scaled by gene expression level. **E**, Heatmap of the main cell types in the murine dataset, based on the level of expression of canonical cell type-specific marker genes. Non-malignant and malignant ETMR cell subpopulations are assigned by grey/black bars. ETMR cells (RG-like, NProg-like, and Nb-like) are named after their non-tumoral counterpart based on their gene expression similarity. Color-coded by cell type (headers) and expression level. **F**, Pearson correlation plots depicting the similarities between malignant and non-malignant cells in the murine dataset, using the full set of genes for each cell type (ref. Fig. 1D). Pearson's product-moment correlation, p-value < 0.0001 for RG-like vs RG, NProg-like vs NProg, and Nb-like vs Nb. **G**, t-SNE plot showing the cell cycle phase in the murine dataset. Color-coded by cell cycle phase. **H**, Heatmap showing that RG-like, NProg-like and Nb-like tumor cells differs in the enrichment of distinct metabolic pathways and in mitotic capacity.

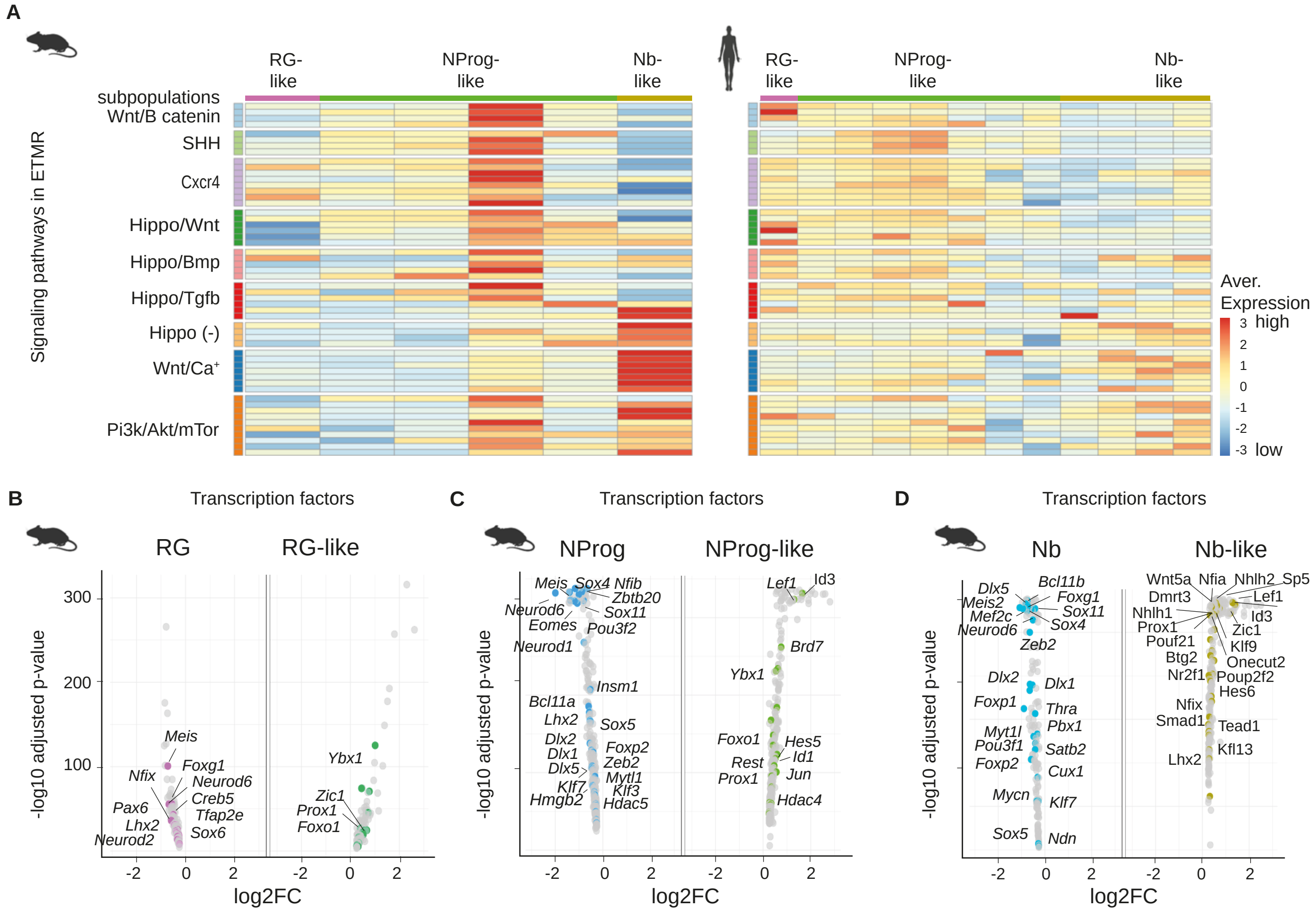

Supplementary Fig. 3. ETMR malignant subpopulations are characterized by distinct signaling pathways and transcription regulation. **A**, Heatmap representation of marker genes from signaling pathways enriched in the ETMR murine subpopulations (left) and validated in the human (right) coun-terparts. Color-coded by gene enrichment score. Row headers define ETMR cell subpopulations (ref. Fig. 1E) and each column under each cell type represents one cellular cluster. **B-D**, Transcription regulation in malignant cells differed from paired healthy cell types of the murine forebrain. Volcano plots descri-bing the top differentially expressed transcription factors and transcription regulators between RG or RG-like cells (B), NProg or NProg-like cells (C), and Nb or Nb-like cells (D). Colored by cell type. The p-value is shown on the y-axis, and the average Log2 fold change (Log2FC) is shown on the x-axis. Source data of **B**, **C** and **D** are provided as a Source Data file. Sample type symbols in **A**-**D** created with Biorender.com

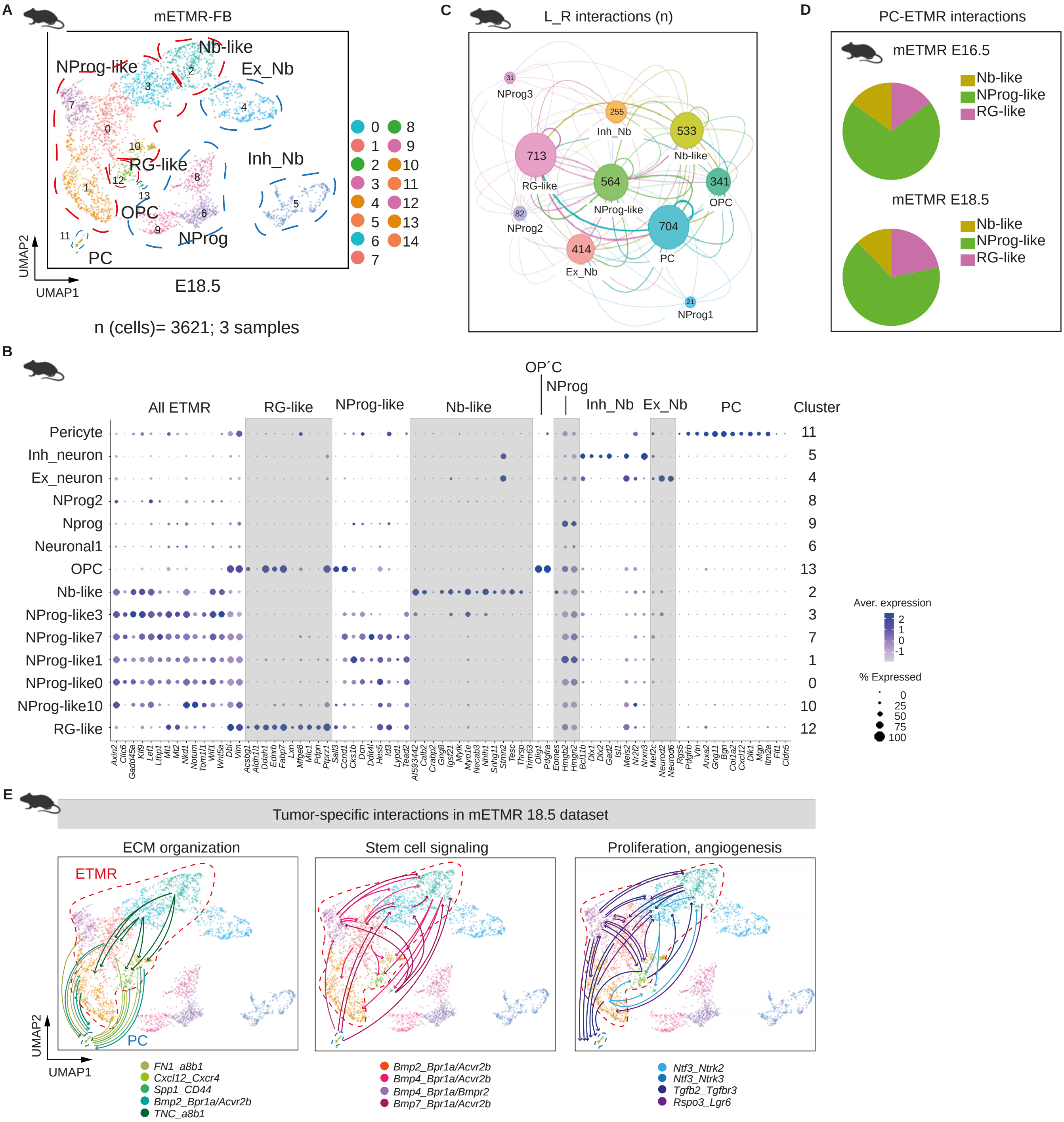

Supplementary Fig. 4. Murine validation cohort confirmed the presence of ETMR subpopulations and PC-ETMR inter-actions via the TME. **A**, UMAP representation of the main cellular lineages of our murine ETMR validation cohort (n = 3 ETMR-harboring forebrains at age E18.5). Color-coded by unsupervised clustering. Dashed lines delineate the clusters repre-senting the main cell types found in the data (ref. Fig. 1C, 1E). Cluster 14 was excluded from further analysis due to the low cell number (< 5 cells). **B**, Cell annotation of ETMR subpopulations and TME. Dot plot description of the marker gene expressi-on for the identified cell types and corresponding clusters. The size of the dots represents the percentage of cells within a cluster expressing the gene. The color intensity shows the average expression level of the indicated gene per cell type. **C**, Network of ligand-receptor interactions predicted by CellphoneDB and visualized by InterCellar. The total number of interactions for each cell type is represented by the size of the circles and specified by numbers. Color-coded by cell type. The thickness of the connecting lines represents the enrichment of L_R pairs between two cell part-ners. **D**, Pie charts comparing the proportional distribution of PC ligands to receptors in RG-like, NProg-like, and Nb-like cells in the original murine cohort and the validation cohort. **E**, Schematic representation of the tumor-specific interactions embedded in the UMAP of the validation cohort. In the central panel (stem cell signaling), interactions involving *BMP2* were omitted for clarity (already represented in the "ECM organization" panel). For interactions involving *BMP7*, the top-scored interactions are plotted. In proliferation/angiogenesis, the interactions with the lowest scores were omitted for clarity. Source data of **D** is provided as a Source Data file. Sample type symbols in **A-E** created with Biorender.com

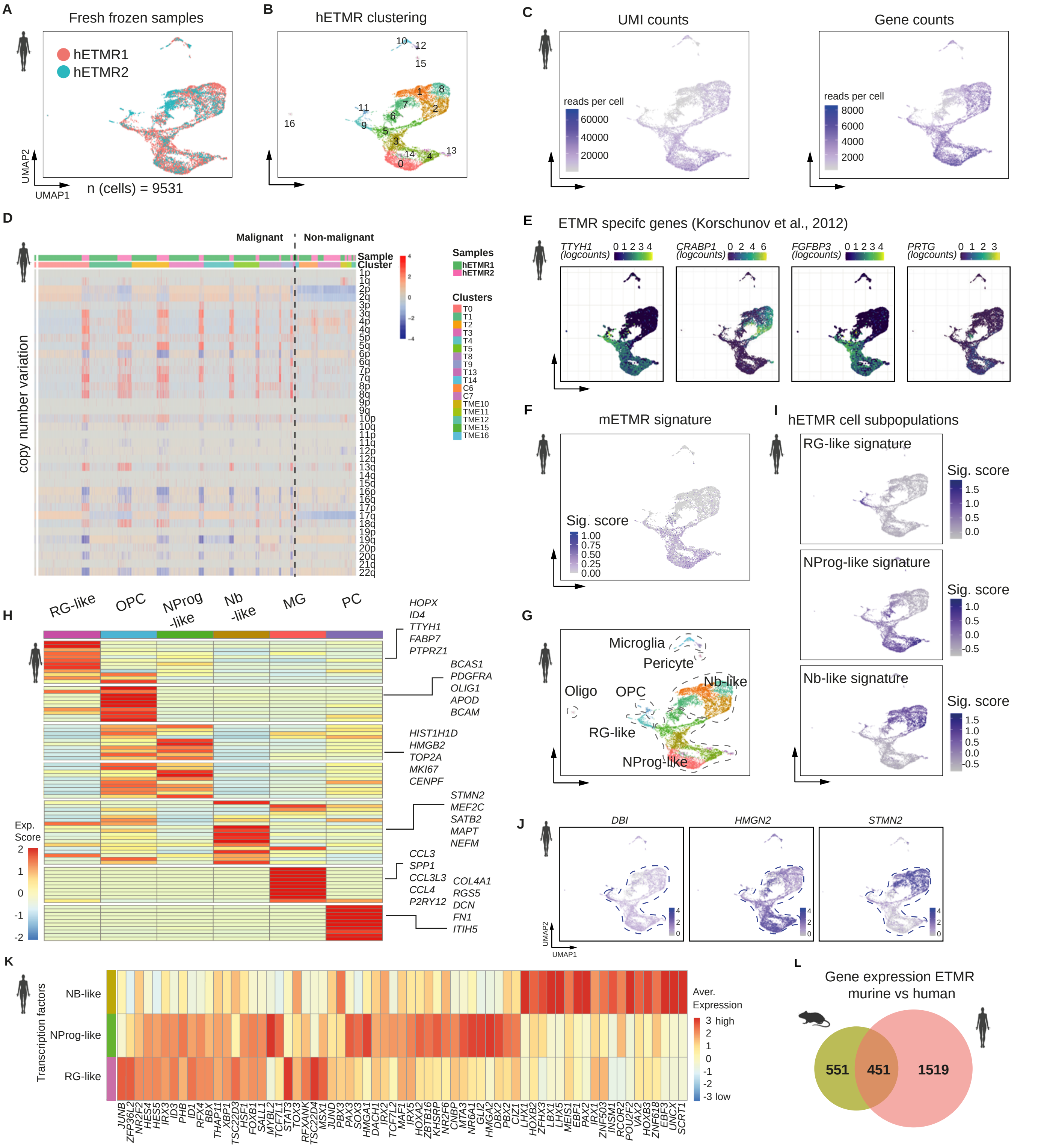

Supplementary Fig. 5. scRNA-seq of human tumors correlated with the murine ETMR subpopulations and TME composition . **A**, UMAP representation of the sample distribution of the two human datasets. Color-coded by sample (n (tumors) = 2); **B**, UMAP depicts the distinct clusters representing the cell types or states defined by unsupervised clustering. Color-coded by unsupervised clustering. **C**, Number of unique molecule identifiers (UMI) and gene counts in the dataset. Cluster 6 and 7 have a relative lower number of reads per cell. Color-intensity indicates the number of reads per cell. **D**, Copy number variation (CNV) plot of the cells from both hETMR samples, defining clusters with malignant (T) and non-malignant cells (TME). Color-code defines chromosome (Chr) gain (red) or loss (blue). Gain of Chr 2 was the hallmark of the malignant cells (Fan et al., 2018). Clusters 6 and 7 (C6, C7) remained undefined, due to their relative lower gene expression. **E**, UMAP representation of ETMR marker genes previously defined by bulk RNA-seq (Korshunov et al., 2012) and enriched in the scRNA-seq dataset. Cluster 6 and 7 were included among the tumor-enriched clusters. Color-coded by gene log counts per cell. **F**, mETMR gene signature represented in the human data. Color-coded by gene signature enrichment score. **G**, UMAP representation of the final annotation of the cell types in hETMR including TME. Color-coded by original clustering. Dashed lines define the corresponding cell types. **H**, Heatmap of the representative marker genes defining the cell type annotation for malignant and non-malignant populations of hETMR tumors. Color-coded by gene enrichment score and cell type. **I**, UMAPs of the hETMR malignant cells by subpopulation (RG-like, NProg-like and Nb-like), assigned based on the complementary approaches of CNV analysis, ETMR marker genes and mETMR signature. Color-coded by gene signature enrichment score. **J**, UMAP depicts the genes *DBI* (RG marker), *HMGN2* (NProg marker), and *STMN2* (Nb marker) embedded in the human clustering. Blue dashed lines surrounds ETMR clusters. Color-scaled by gene expression level. **K**, Transcription regulation in human malignant cells. Heatmap with the most differentially expressed transcription factors and regulators in human ETMR subpopulations. Color-coded by enrichment score. **L**, Venn diagram depicting the correspondence between murine and human ETMR tumors gene expression, considering all up-regulated genes in ETMR cells from both species. Color-coded by species: murine (green) and human (red)

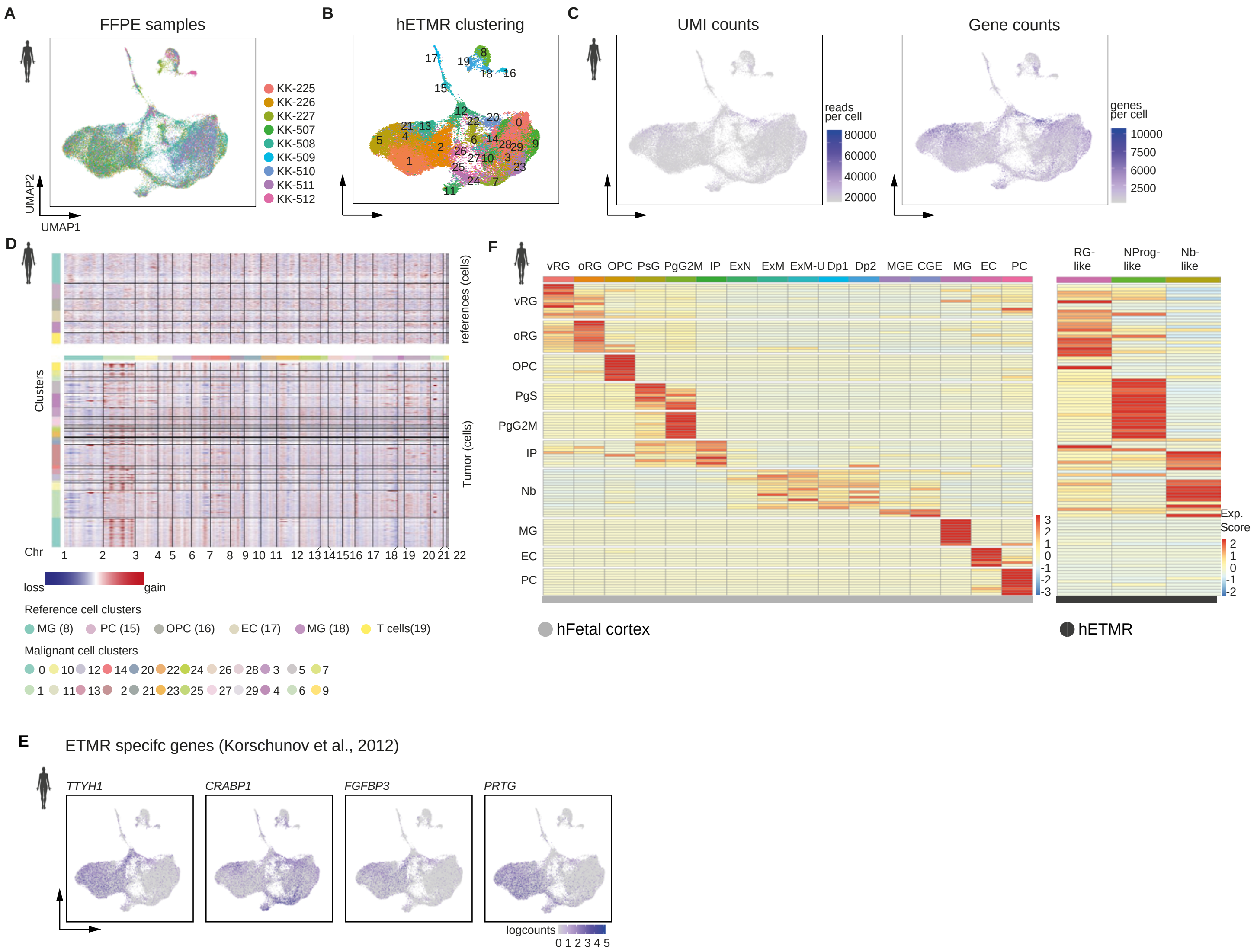

Supplementary Fig. 6. FixedRNA-seq of human tumors validate cell annotation of scRNA-seq. **A**, UMAP representation of the FFPE samples processed for fixedRNA-seq (10xGenomics) (n samples = 9). Color-coded by sample; **B**, UMAP depicts the distinct clusters representing the cell types or states defined by unsupervised clustering. Color-coded by unsupervised clustering. **C**, Number of unique molecule identifiers (UMI) and gene counts in the dataset. Color-intensity indicates the number of reads/-ge-nes per cell. **D**, Copy number variation(CNV) plot of cells from all samples. Color-code defines chromosome (Chr) gain (red) or loss (blue). Gain of Chr 2 was the hallmark of the malignant cells (Fan et al., 2018). **E**, UMAP representation of ETMR marker genes previously defined by bulk RNA-seq (Korshunov et al., 2012) and enriched in the scRNA-seq dataset. Color-coded by gene log counts per cell. **F,** Cell type-specific marker genes extracted from scRNA-seq of human fetal neocortex at mid-ge-station (17-18 weeks)[23] were used to annotate the original dataset and our human ETMR malignant dataset. Tumor cells were named after their non-tumor counterpart. Abbreviations: oRG: outer radial glia; vRG: ventral radial glia; OPC: oligoprogenitor cells; PgS: progenitors in S-phase; PgG2M: progenitors in G2/M-phase; IP: intermediate progenitor; ExN: migrating excitatory; ExM: maturing excitatory; ExM-U: maturing excitatory upper enriched; Dp1: excitatory deep layer 1; Dp2: excitatory deep layer 2; MGE: medial ganglionic eminence-derived interneurons; CGE: cortical ganglionic eminence-derived interneurons; NProg: neuroprogenitor; Nb: neuroblast; MG: microglia; PC: pericytes: EC: endothelial cells; Sample type symbols in **A**, **C-E** created with Biorender.com

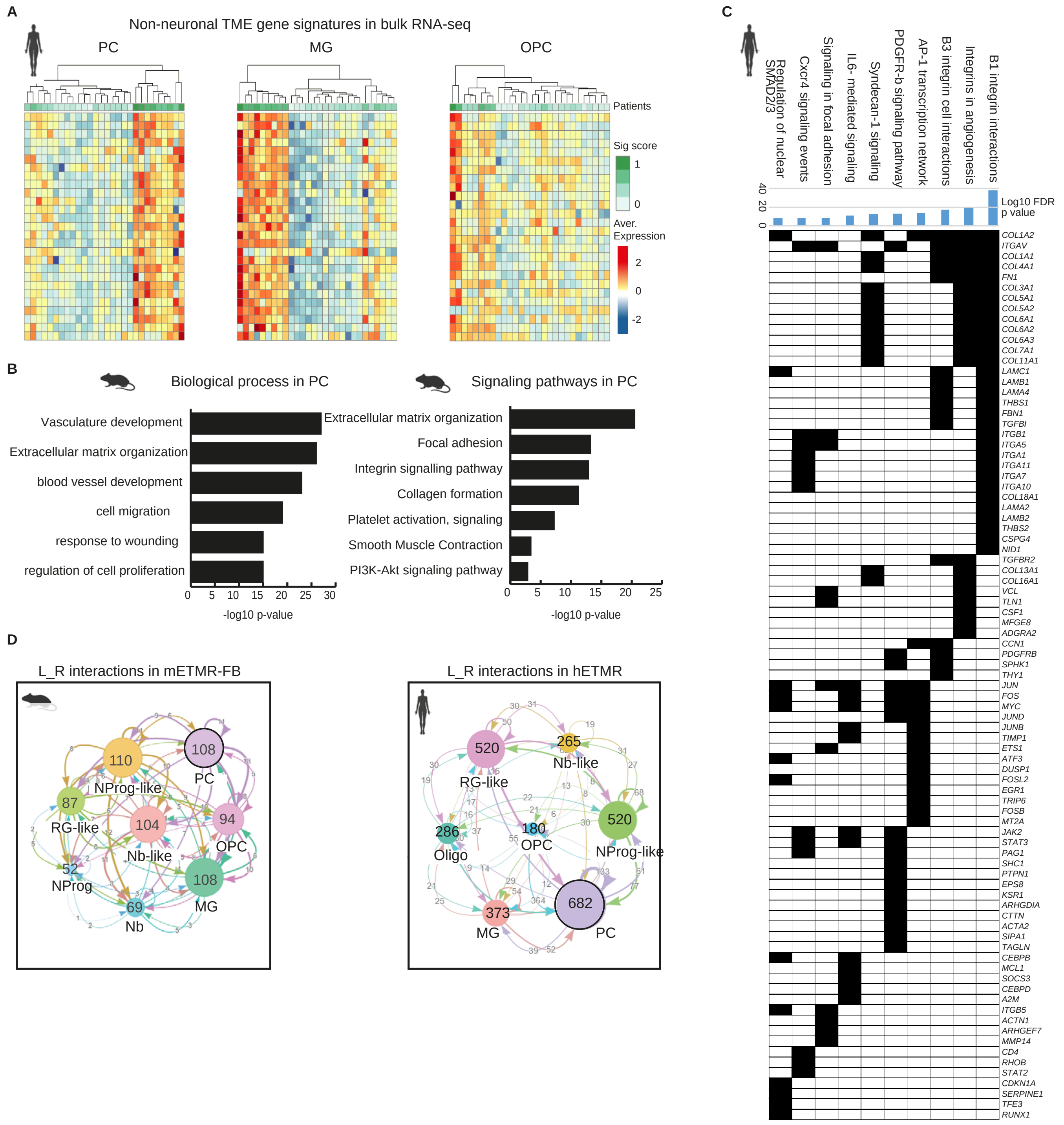

Supplementary Fig. 7. PC relevance to ETMR TME is supported by bulk RNA sequencing and L_R interactions analysis of murine and human tumors. A, Unsupervised clustering of 28 human ETMR samples (bulk-RNA sequencing from Lambo et al., 2019) based on the differential enrichment for a set of representative marker genes of pericytes (PC), microglia (MG), and oligopro-genitor cells (OPC). **B**, Bar graph depicting top ranked (p-value based) GO terms and signaling pathways upregulated in pericytes in the murine scRNA-seq dataset. **C**, Gsea canonical pathways (PID collection) analysis from top 500 differential-ly expressed genes in PCs from the human snRNA-seq dataset. Pathway-associated genes and false discovery rate(FDR) q-value for every predicted signaling are displayed. **D**, Network of total ligand-receptor interactions between ETMR cells and relevant cell populations of the tumor microenvironment. Numbers represent total interactions per cell type, generated by and visualized with InterCellar (Tormo, 2018; Interlandi, 2022). The two panels represent the network in the murine dataset (mETMR-FB) (left) or the human dataset (hETMR) (right) (Ref. Fig. 1C, 1D). Color-coded by cell type.The thickness of the connecting lines represents the enrichment of ligand-receptor (L_R) pairs between two cell partners. Source data of **D** is provi-ded as a Source Data file. Sample type symbols in **A-D** created with Biorender.com

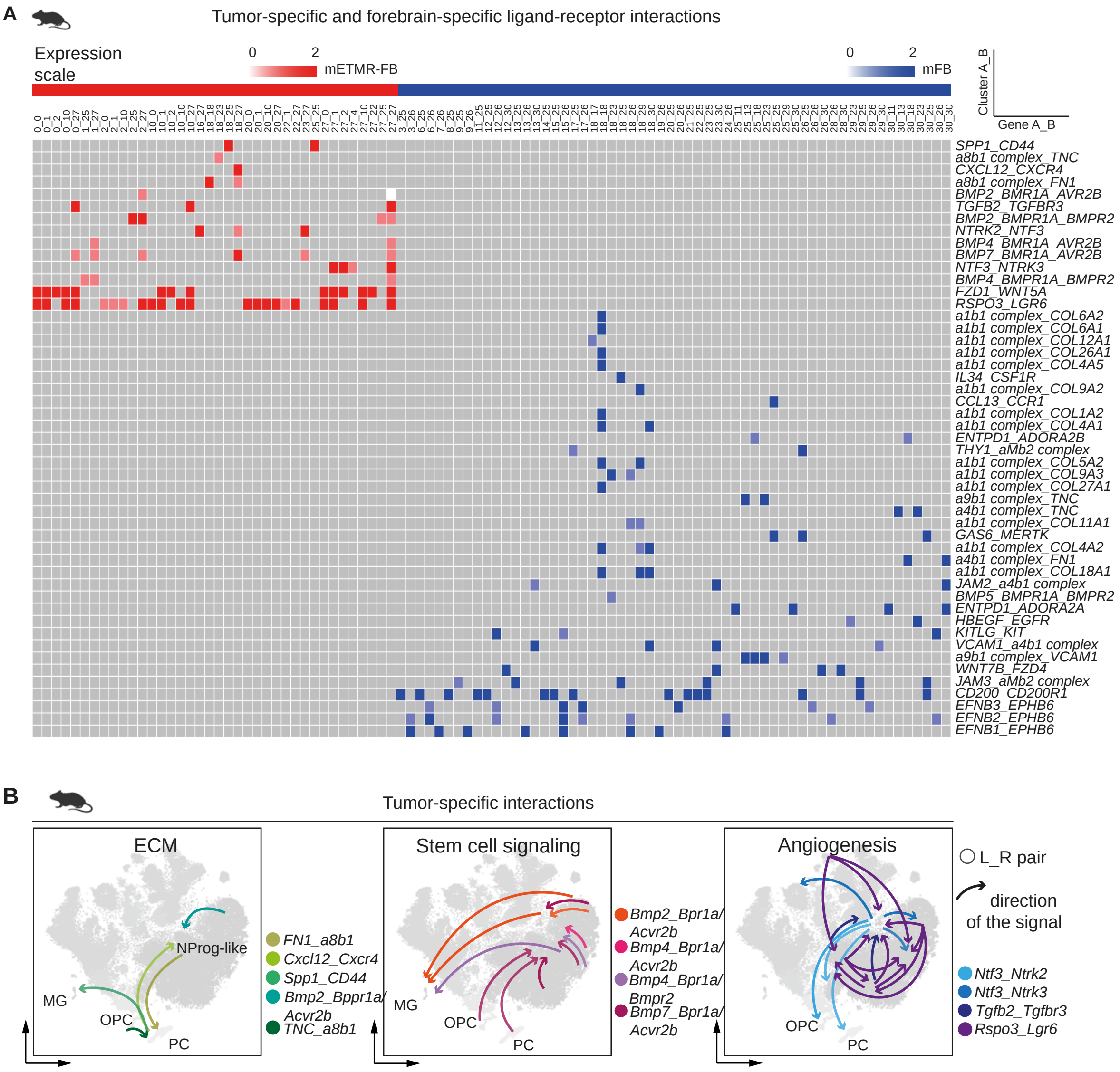

Supplementary Fig. 8. Tumor-specific interactions revealed cell-cell communications related to ECM organization, stemness, and angiogenic growth in the TME. A, Heatmap describes the unique (L_R) interactions in the murine ETMR-harboring (mETMR-FB) or wildtype murine (mFB) forebrains. Columns represent cluster-cluster (clusterA_clusterB) interacting pairs and rows indicate molecules (ligand A_receptor B) involved in the unique interactions. Color-coded by scaled CellPhoneDB interaction score per condition: mETMR-FB specific (red) and mFB specific (blue). Grey denotes absence of interactions. **B**, Scheme of L_R interaction dynamics in the murine dataset, embedded in the UMAP (cells in grey). Cell types and molecules participating in tumor-specific L_R interactions classified into three main branches: ECM organization, stem cell signaling, and proliferation/angiogenesis. The arrows define the direction of the interaction (A_B): the arrow base represents a ligand A in cell A and the arrowhead represents a receptor B in cell B. Color-coded by ligand_receptor (A_B) molecule pairs. Sample type symbols in **A**, **B** created with Biorender.com

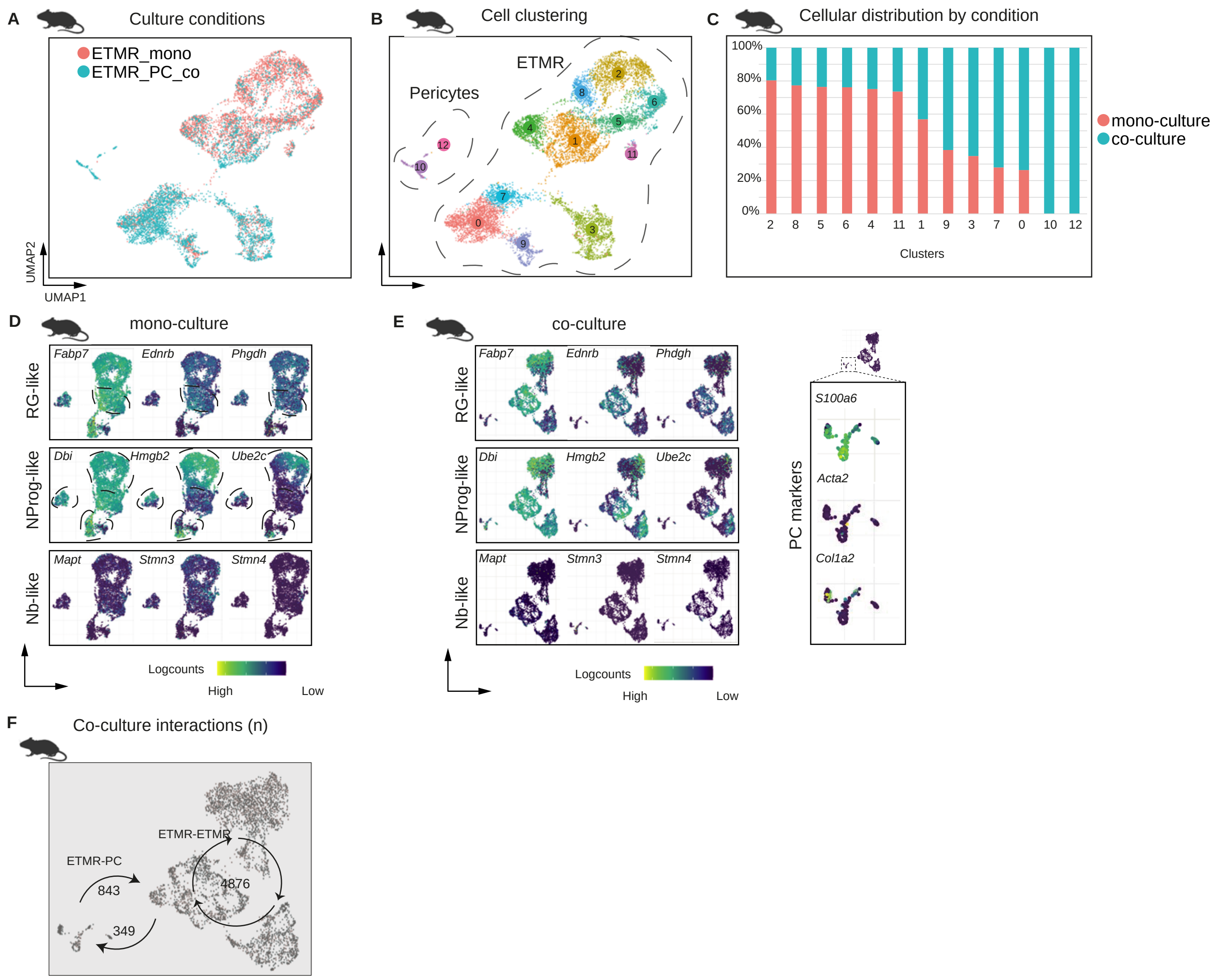

Supplementary Fig. 9. Co-culture of pericytes with ETMR impacted the tumor gene expression profile. **A**, UMAP repre-sentation of the integrated datasets of the ETMR mono-culture (control) and the ETMR-PC co-culture systems. Cells are color-coded by culture conditions (mono vs co-culture). **B**, UMAP defining the unsupervised clustering of the identified cell types in the dataset. Dashed lines delineate ETMR cells and pericytes. Color-coded by original clustering. **C**, Bar graph showing the relative contribution of cells from each condition to the cluster composition. Color-coded by condition, as in (A). **D**, UMAP plots of cells in the mono-culture conditions, depicting the representative marker genes of the ETMR subpopulations RG-li-ke, NProg-like, and Nb-like, color-coded by gene log counts. **E**, UMAP plots of the cell in co-culture conditions, showing the repre-sentative marker genes of the ETMR subpopulations and PC in the dataset and color-coded by gene log counts. **F**, Sche-matic representation of the number of ligand-receptor interactions of the co-culture condition, embedded in the UMAP and predicted by CellphoneDB. The number of ETMR-PC interactions and the number of ETMR-ETMR interactions are described. Arrows indicate the direction of the interactions. Sample type symbols in **A**-**F** created with Biorender.com

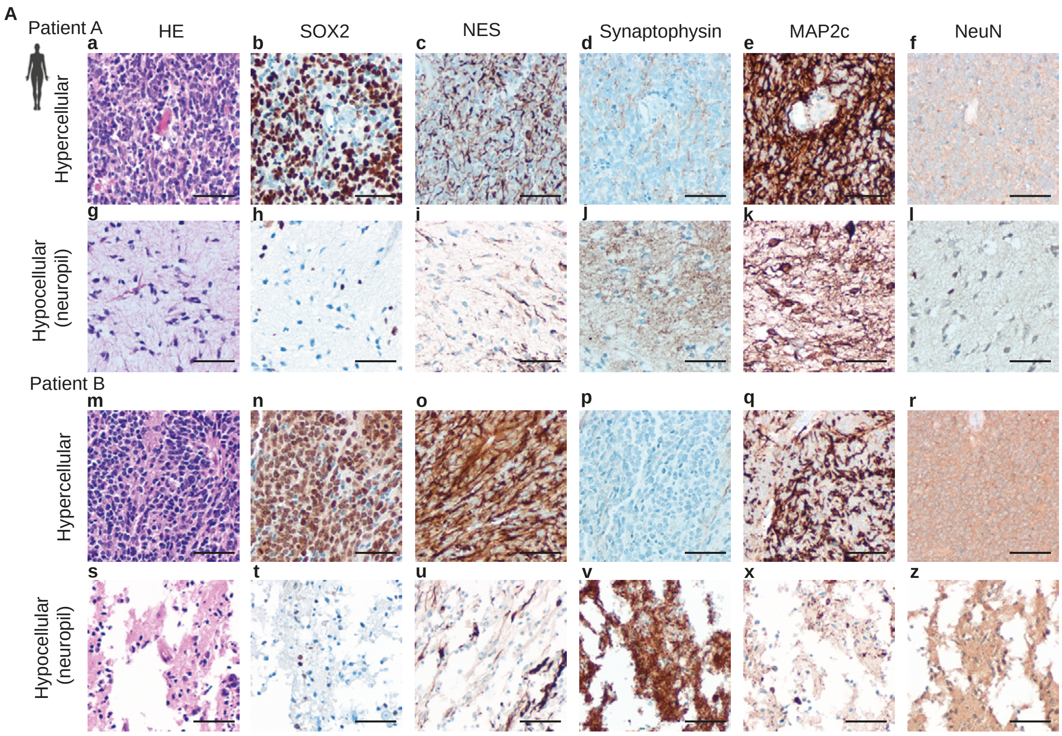

Supplementary Fig. 10. ETMR hypercellular regions are enriched in stem cell markers. **A**, (a-l; m-z) Immunohistochemical profile of hypercellular (cell dense) and hypocellular (neuropil) regions of two distinct human ETMR primary tumors (patient A; B); 20x magnification, scale bar = 50 µm. (a-f; m-r) Hypercellular regions were characterized by high cell density in H&E (a; m) and stained positive for the markers SOX2 (b; n), Nestin (NES) (c; o) and MAP2c (e; q), while showing no expression of neuronal markers NeuN (f; r) and very weak expression of synaptophysin (d; p). (g-l; s-z) Hypocellular (neuropil) regions displayed low cell density in H&E (g; s), mostly lacked expression of SOX2 (h; t) and Nestin (i; u) and were enriched in synaptophysin (j; v) with diffuse expression of MAP2c (k; x) and NeuN (l; z). Sample type symbol created with Biorender.com

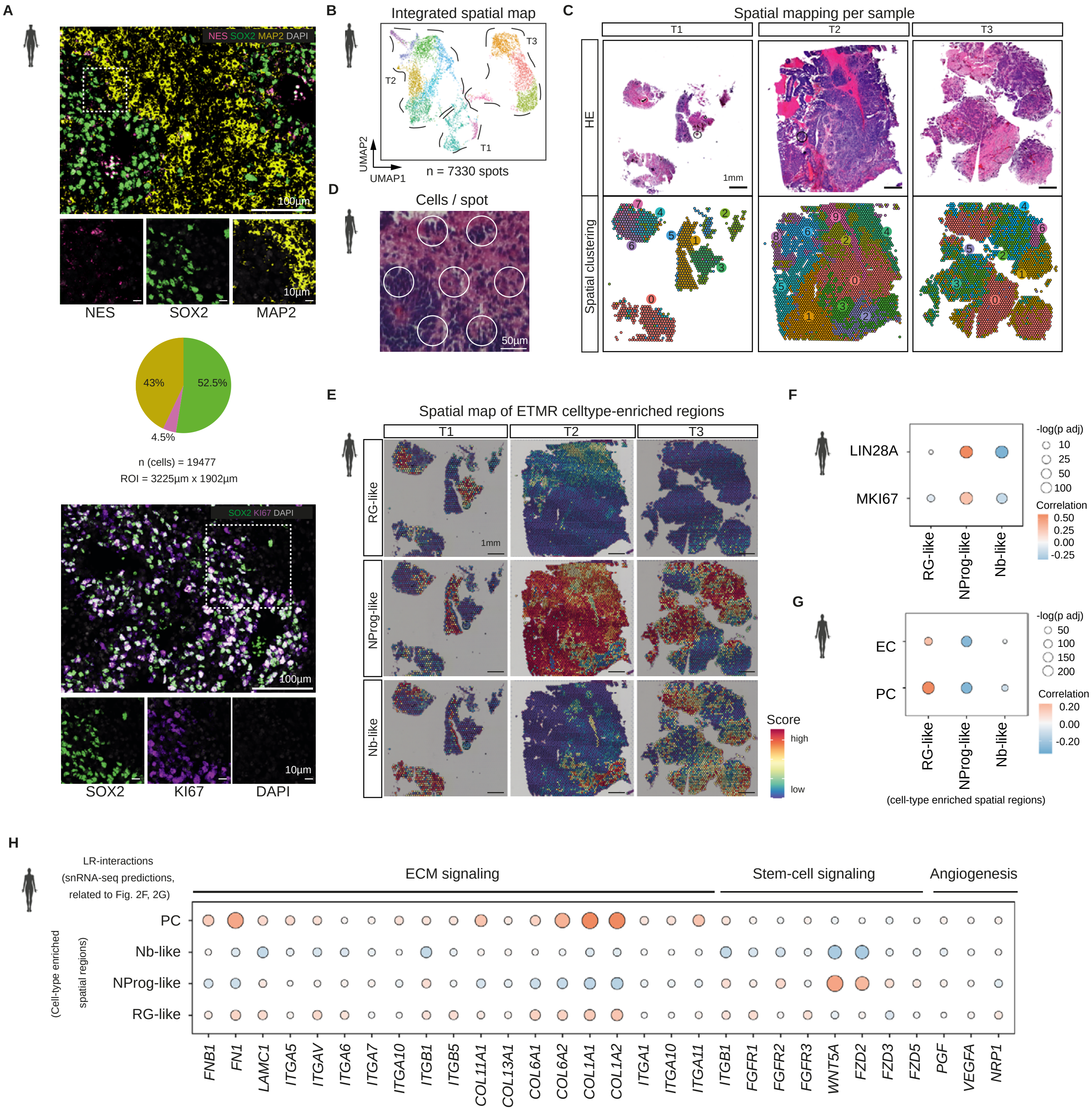

Supplementary Fig. 11. ETMR subpopulations were spatially organized, forming distinct niches. **A**, Immunofluorescene imaging of RG-like (NES$_{high}$ / SOX2$_{+}$), Nprog-like (NES$_{low-}$ /SOX2$_{+}$) and Nb-like (SOX2$_{-}$/MAP2$_{+}$) cells in the upper panel, with description of cell-type relative proportion among 19477 segmented cells; In the lower panel, KI67 and SOX2 expression of the same ROI. Imaging of a representative ETMR human tumor area (ROI = 3225 µm x 1902 µm) pathologically assigned. Scale bar = 100µm and 10µm. **B**, Visium (10x Genomics) spatial transcriptomics UMAP representing the integrated clustering of three slides with human chemo-naive ETMR tumors from distinct patients (T1, T2, T3). Cluster assignment by sample is depicted in dashed lines. **C**, Panel of H&E staining and corresponding Seurat spatial transcriptomic clustering for each individual sample. Color-coded and numbered by cluster composition of the respective sample. scale bars = 1 mm. **D**, Cell number variation per spot in distinct tumor areas. Cell nuclei assigned by hematoxylin staining. White circles mark the area covered by each spot subjected to pooled transcriptomic analysis. 50x enlargement of a representative area of T2, scale bar = 50 µm. **E**, Spatial transcriptomic maps of the samples T1, T2 and T3 defining RG-like-, NProg-like- and Nb-like-enriched tumor regions, by sample. Color-coded by gene set enrichment score. scale bars = 1 mm. **F**, LIN28A and MKi67 correlation with distinct celltype-enriched regions in the spatial integrated dataset. Color-coded by correlation score. **G**, Pericyte (PC) and endothelial cell (EC) gene expression correlation with ETMR celltype-enriched spatial regions in the integrated dataset. Color-coded by correlation score. **H**, Spatial correlation scores of ligand and receptors from L-R interactions described in Fig. 2F, 2G. Sample type symbols in **A-H** created with Biorender.com

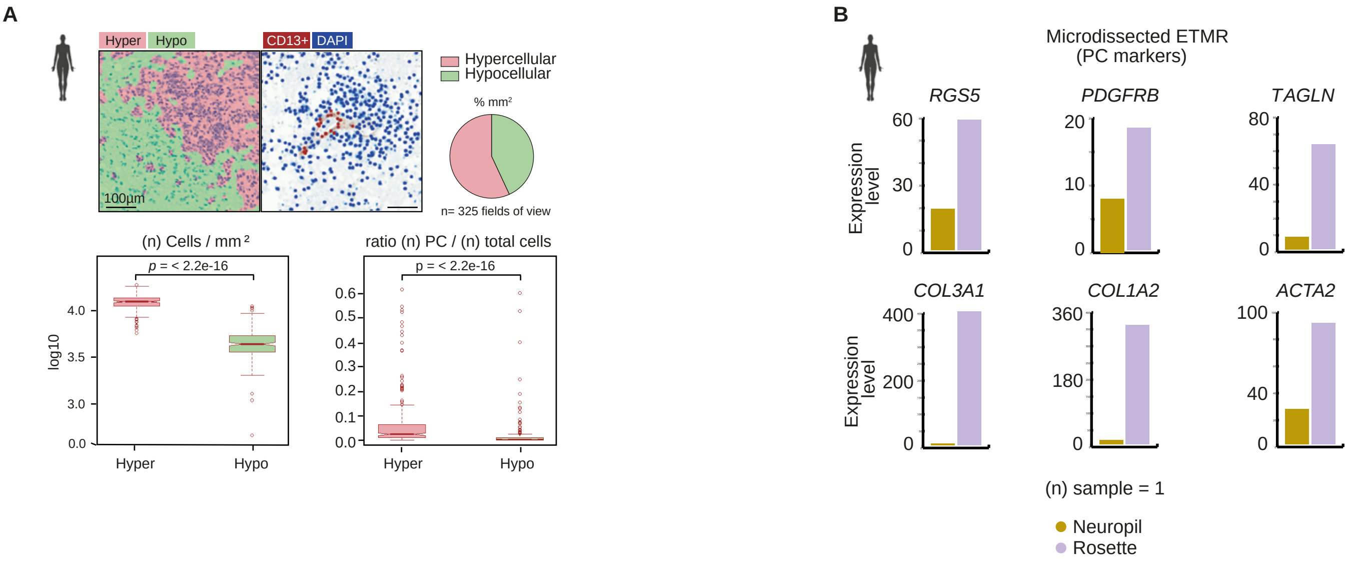

Supplementary Fig. 12. PCs are enriched in ETMR hypercellular regions, which correlate with stem cell-like cell fates. **A**, Quantitative immunohistochemistry (IHC) of human ETMR tumors (n slides = 4). The left panel shows representative image tissue segmentation, defining regions of interest (ROIs) of ETMR IHC sections as hypercellular (Hyper, pink) regions and hypocellular (Hypo, green) regions (see Fig.3): the right panel depicts the assignment of all DAPI+ cells (blue) and CD13+ PC (red) for further quantification. Scale bar = 100µm. Proportional contribution of Hyper and Hypo regions to ETMR compo-sition depicted in a pie chart of all fields of view analyzed by IHC. Box plots display the total cell density (left) and the ratio of PC per all other cells (right) in the Hyper and Hypo regions (n samples = 4; 325 fields of view non-overlapping and manually assigned, field of view = 669 µm x 500 µm). Boxes represent the median (middle) and interquartile ranges (upper /lower hinges); whiskers show 1.5xIQ. Data points 1,5x more than the length of the box away from the box were considered outliers. One-sided Wilcoxon rank sum test with continuity correction. Source data is provided as Source Data file. **B**, Bulk RNA-sequencing of a published ETMR sample microdissected into rosettes vs neuropil ROI (n sample = 1). Bar graph represents PC-specific marker genes, depicting their differential LogFC in the distinct microdissected regions. Source data at Lambo et al., (2019)7.Sample type symbols in **A**, **B** created with Biorender.com

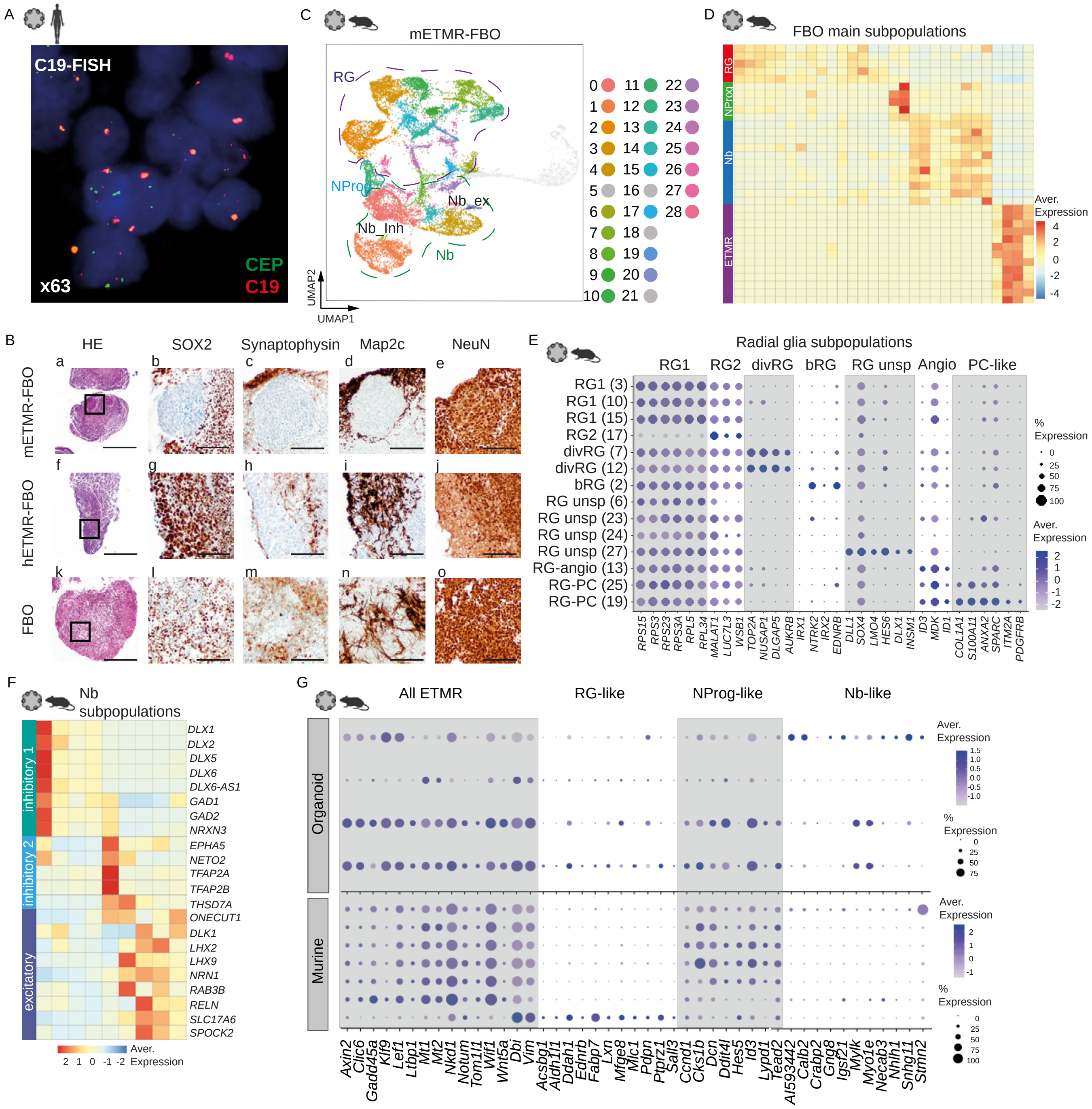

Supplementary Fig. 13. mETMR-FBO recapitulated mETMR cellular heterogeneity. **A**, Fluorescence in situ hybridization (FISH) confirming C19MC amplification in tumor regions of hETMR-FBO (CEP = Chromosome enumeration probes); 63x magnification; n = 20. **B**, Profile of neuronal markers in FBO, mETMR-FBO, and hETMR-FBO IHC staining. (a, f, k) H&E staining of the histological sections chosen for further analysis. Areas amplified for further descriptions are depicted by the squares (4x magnification, scale bar = 250 µm); (b-e, g-j, l-o) 20x magnification (scale bar = 50 µm) view of the areas selected for description; (b-e) Tumoral areas in mETMR-FBO were negative for SOX2 (b), Synaptophysin (c) and MAP2c (d), although strongly positive for the neuronal marker NeuN (e); (g-j) Cellular dense malignant regions in hETMR-FBO highly expressed SOX2 (g), with sparse positivity for synap-tophysin (h), strong expression of MAP2c (i) but no nuclear expression of NeuN in comparison to mETMR-FBO and the FBO background (j). (l-o) Healthy FBO were characterized by moderate and diffuse expression of SOX2 (l), Synaptophysin (m) and MAP2c (n) All cells stained positive for NeuN (o). **C**, UMAP represents the 29 cellular states identified in the integrated clustering of the mETMR-FBO (n =3 biological replicates) and FBO without ETMR cells (control, n = 2 technical replicates) and plots the cell types or subtypes defined by unsupervised clustering. Color-coded and numbered by cluster, with murine ETMR cells in grey. Dashed lines depicting the major cell types in FBO. **D**, Heatmap of marker genes defining ETMR and the metaclusters of the major FBO cell types identified in the dataset at thirty days post co-aggregation (D30) (ref. Fig. 4E-G). Columns represent individual clusters. Rows represent marker genes. Color-coded by average expression level. **E**, Dot plot representing the marker genes of the RG subpopulations identified in the dataset. The corresponding cluster numbers are presented in parentheses (). The size of the dots represents the percentage of cells within a cluster expressing the gene. The color scale shows the average expression level of the indicated gene per cell subtype. **F**, Heatmap of the top marker genes defining the Nb subpopulations identified in the FBO. Columns represent distinct clusters as in (C). Color-coded by average expression level. **G**, Dot plot representing the marker genes for the 3 malignant subpopulations in murine ETMR and mETMR-FBOs. The color scale indicates the average expression level of the indicated gene per cell subtype. Sample type symbols in **A**, **C-G** created with Biorender.com

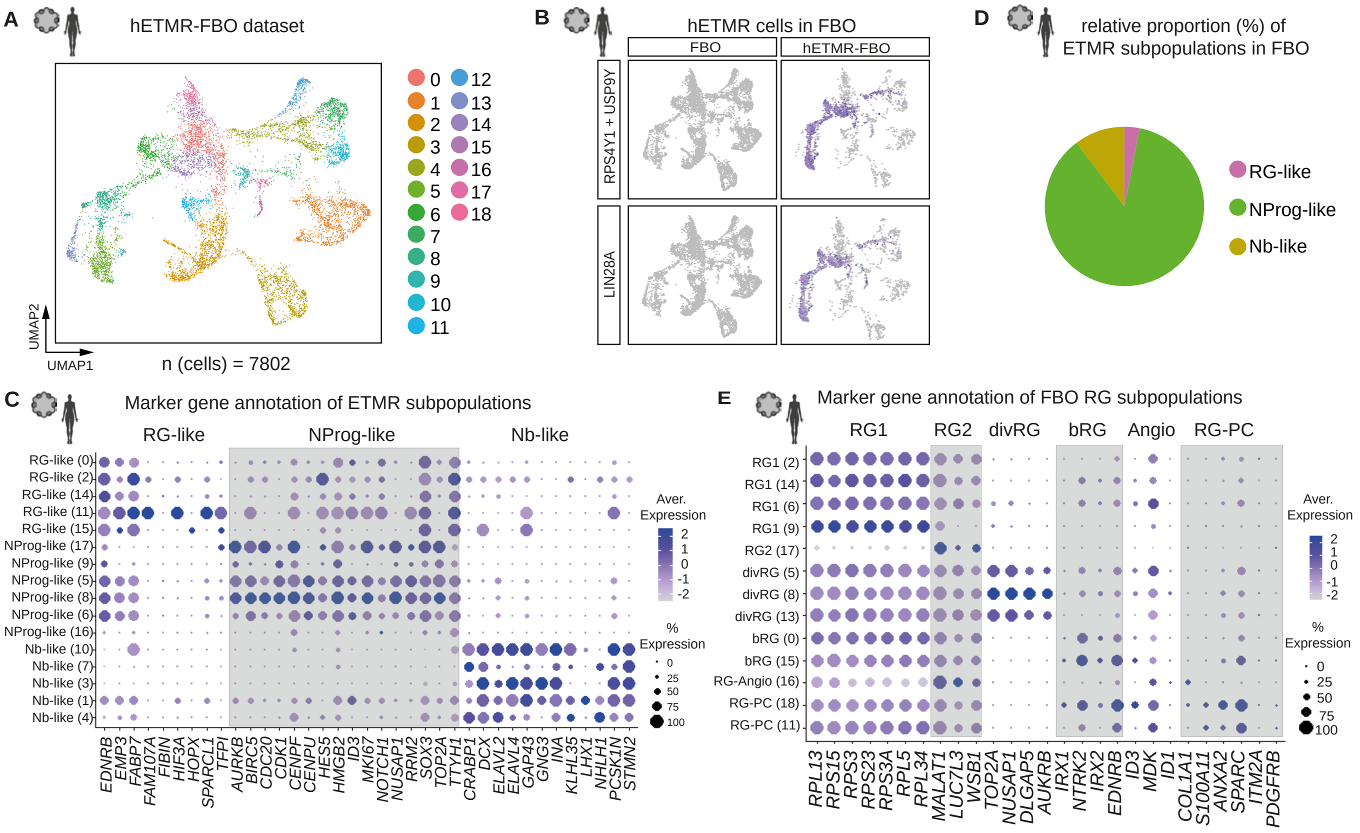

Supplementary Fig.14. hETMR-FBO recapitulated hETMR cellular heterogeneity. A , UMAP representing the 19 cell states defined by unsupervised clustering and corresponding to cell types or sub-types in the hETMR-FBO dataset. Color-coded by cluster. **B**, Panel of UMAPs showing the assignment of ETMR cells in the dataset, based on the expression level of Y-Chro-mosome gene RPS4Y1 and LIN28A. **C**, Dot plot of major marker genes defining ETMR subpopulations in the dataset. The corresponding cluster numbers are presented in parentheses. The size of the dots represents the percentage of cells within a cluster expressing the gene. The color scale shows the average expression level of the indicated gene per cell subtype. **D**, Pie chart depicting the proportional enrichment of RG-like, NProg-like, and Nb-like cells to cellular ETMR composition. **E**, Dot plot of marker genes defining the non-malignant RG subpopulations found in the dataset. Sample type symbols in **A-E** created with Biorender.com

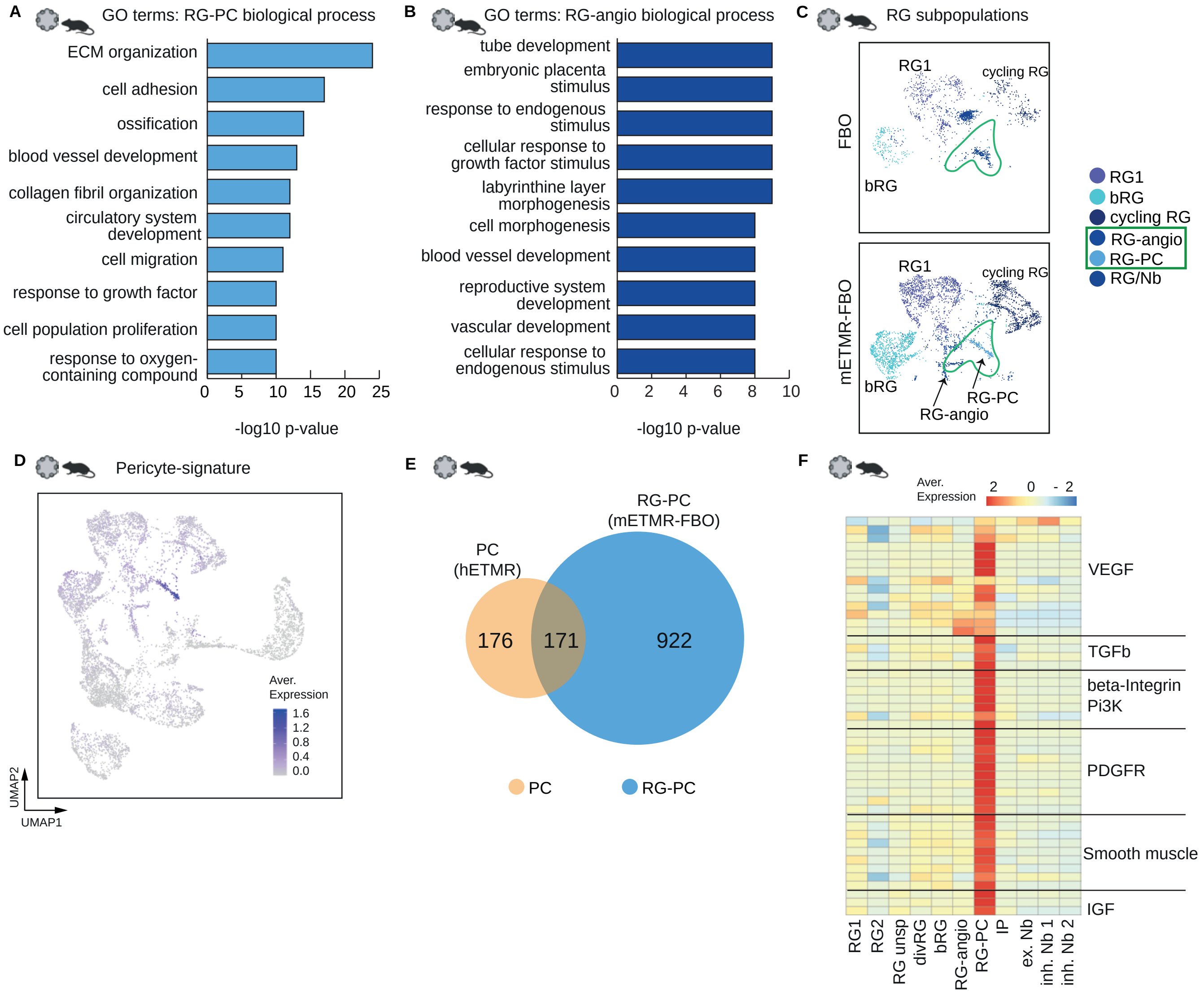

Supplementary Fig. 15. Characterization of RG-derived Angio and PC cells. **A**, **B**, Bar plots depicting the most significant GO terms enriched in the RG-derived PC clusters (RG-PC) (A) and the RG-angio cluster (B), based on their log10 p-value. GO terms were predicted by ToppGene database. Color-coded by cell type as original clustering (ref. Fig. 4F). **C**, UMAPs of the RG clusters, showing the enrichment of RG-angio and RG-PC cells in the FBO and mETMR-FBO datasets. Color-coded by cell type. RG-angio and RG-PC cells are highlighted in green. **D**, UMAP showing the enrichment score of PC gene signature generated from the scRNA-seq of the primary human ETMR. **E**, Venn diagram depicting the gene expression correspondence between the PC cells from the primary human ETMR dataset and the RG-PC cluster from the FBO. The diagram highlights the overlap between the differentially upregulated genes of the RG-PC and the PC from the primary hETMR. Color-coded by cell type. **F**, Heatmap depicting the most enriched signaling pathways in RG-PC cells, predicted by ToppGene. Color-coded by expression level. Sample type symbols in **A-F** created with Biorender.com

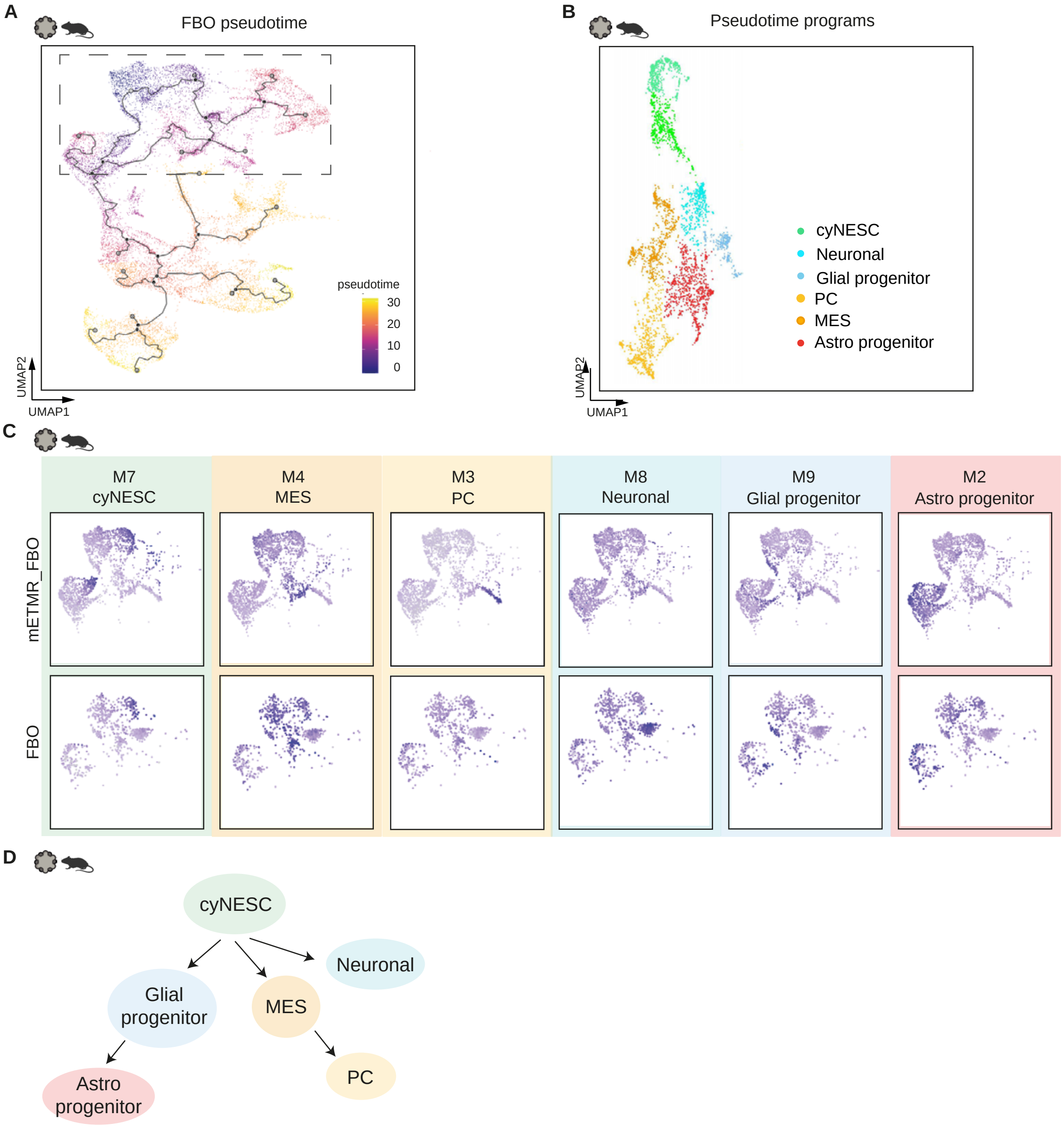

Supplementary Fig. 16. Cell fate commitment of RG cells in mETMR-FBO revealed neuronal, astroglial, and mesen-chy-mal differentiation. **A**, UMAP showing the predicted pseudotime trajectory for all the cells in the dataset. RG cells, highlighted by dashed lines, were selected for further pseudotime analysis. Color-coded by pseudotime. **B**, Pseudotime gene modules corres-ponding to distinct biological programs along the trajectory of the RG cells. Each dot corresponds to a gene. Murine and ribosomal genes were excluded for clarity. Color-coded by gene module and corresponding biological program. **C**, Panel depicting the gene enrichment of the pseudotime biological programs along the distinct RG cells, separated by FBO (cont-rol) vs mETMR-FBO. Color-coded by the corresponding program. **D**, Scheme describing the lineage trajectory from cyNESC program leading to generation of neuronal-, glial-, and mesenchymal-committed RG cells. Color-coded as in (c). Abbreviations: cyNESC: cycling neuroepithelial stem cells; MES: mesenchymal cells; PC: pericytes; Astro: astrocytes. Sample type symbols in **A-D** created with Biorender.com

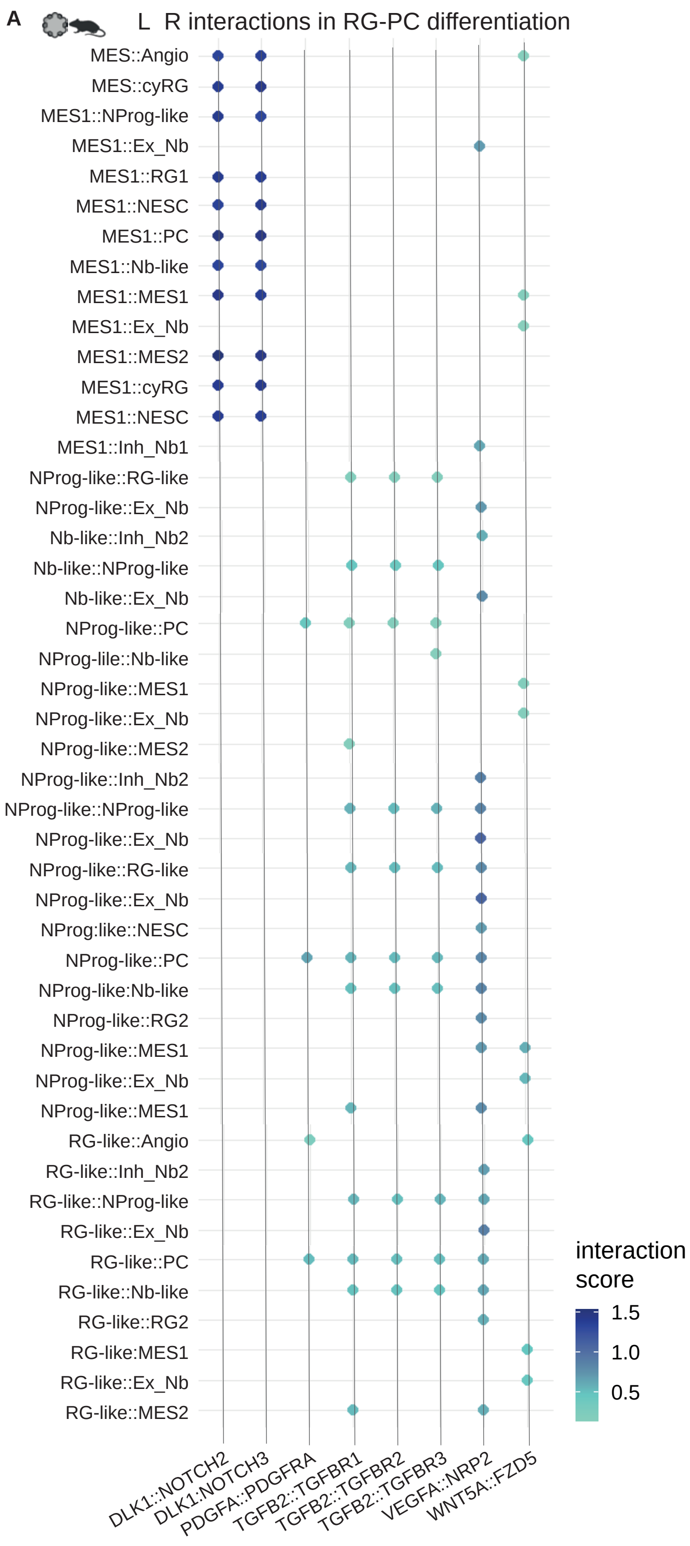

Supplementary Fig. 17. Cell-cell interactions involved in the RG to PC lineage differentiation. **A**, InterCellar dot plot of L_R interactions related to the signaling pathways enriched in the RG to PC lineage differentiation and summarized schematically in Fig. 5F-H). L_R molecule pairs are described in the x-axis. Cell pairs related to the signaling pathways involved in the lineage switch process were selected and depicted in the Y axis. Cell A_ secretes ligand A_ and cell _B receives signals through receptor _B. Color-coded by L_R expression score. Sample type symbols in **A** created with Biorender.com

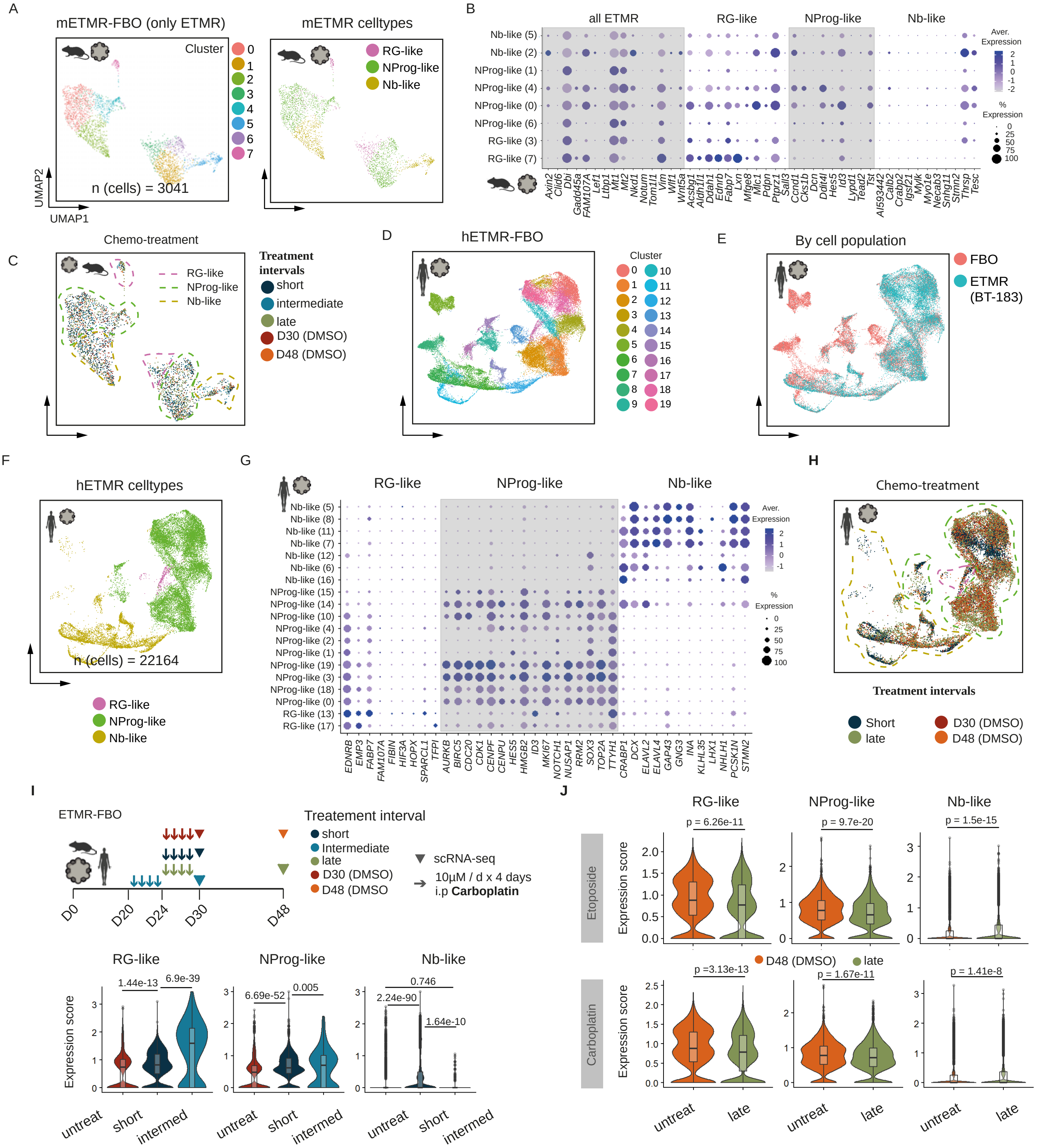

Supplementary Fig. 18. Cellular annotation of the scRNA-seq from murine and human ETMR-FBO treated with carboplatin or etoposide. **A**, UMAPs of murine ETMR cells in the integrated carboplatin- and etoposide-treated mETMR-FBO dataset of organoids harvested for scRNA-seq at distinct treatment intervals (short, intermediate and late; see Fig. 6A). Unsuper-vised clustering (left panel) segregates mETMR cells in 8 clusters, corresponding to the three mETMR cell subpopulations (right). **B**, Marker gene annotation of mETMR cell types depicted in a dot plot. The size of the dots represents the percentage of cells within a cluster expressing the gene. The color scale shows the average expression level of the indicated gene per cell subty-pe. **C**, UMAPs of the chemo- and DMSO-treated mETMR cell subpopulations. Single cells are color-coded by treatment interva-ls. Dashed lines delineate mETMR subpopulations. **D-E**, Unsupervised clustering of the chemo-treated hETMR-FBO represen-ting 20 cellular states (D) corresponding to FBO (female) and hETMR (male) cell subpopulations, separated using Y-Chromo-some specific genes (E). **F**, UMAP of cellular annotation of hETMR cell subpopulations in hETMR-FBO. **G**, Marker genes defining the assignment of the hETMR cell subpopulations found in the dataset. **H**, UMAPs of the chemo- and vehicle-tre-ated hETMR cell subpopulations. Single cells are color-coded by treatment schedule, as in (C). Dashed lines delineate hETMR subpo-pulations. **I**, *Top*: Carboplatin treatment schedule of both mETMR-FBO (n = 12) and hETMR-FBO (n samples = 8). Organoids were exposed to carboplatin (10 µM) or DMSO as a control. Timepoints as per Fig. 6A. *Bottom*: Violin plots show expression scores of ETMR celltype-specific gene signatures comparing D30 (DMSO), short and intermediate intervals in integrated dataset (n = 2 biological replicates / treatment, n = 8204 cells). **J**, Expression scores of ETMR celltype-specific gene signatures at late interval compared to D48 (DMSO) control for carbopatin (n = 10.114 cells) and etoposide (n = 8415 cells). For **I** and **J**, Kruskal-Wallis statistical test was applied for multi-group comparisons, Wilcoxon rank sum test for inter-group comparisons, FDR-corrected p-values are displayed. Sample type symbols in **A-J** created with Biorender.com

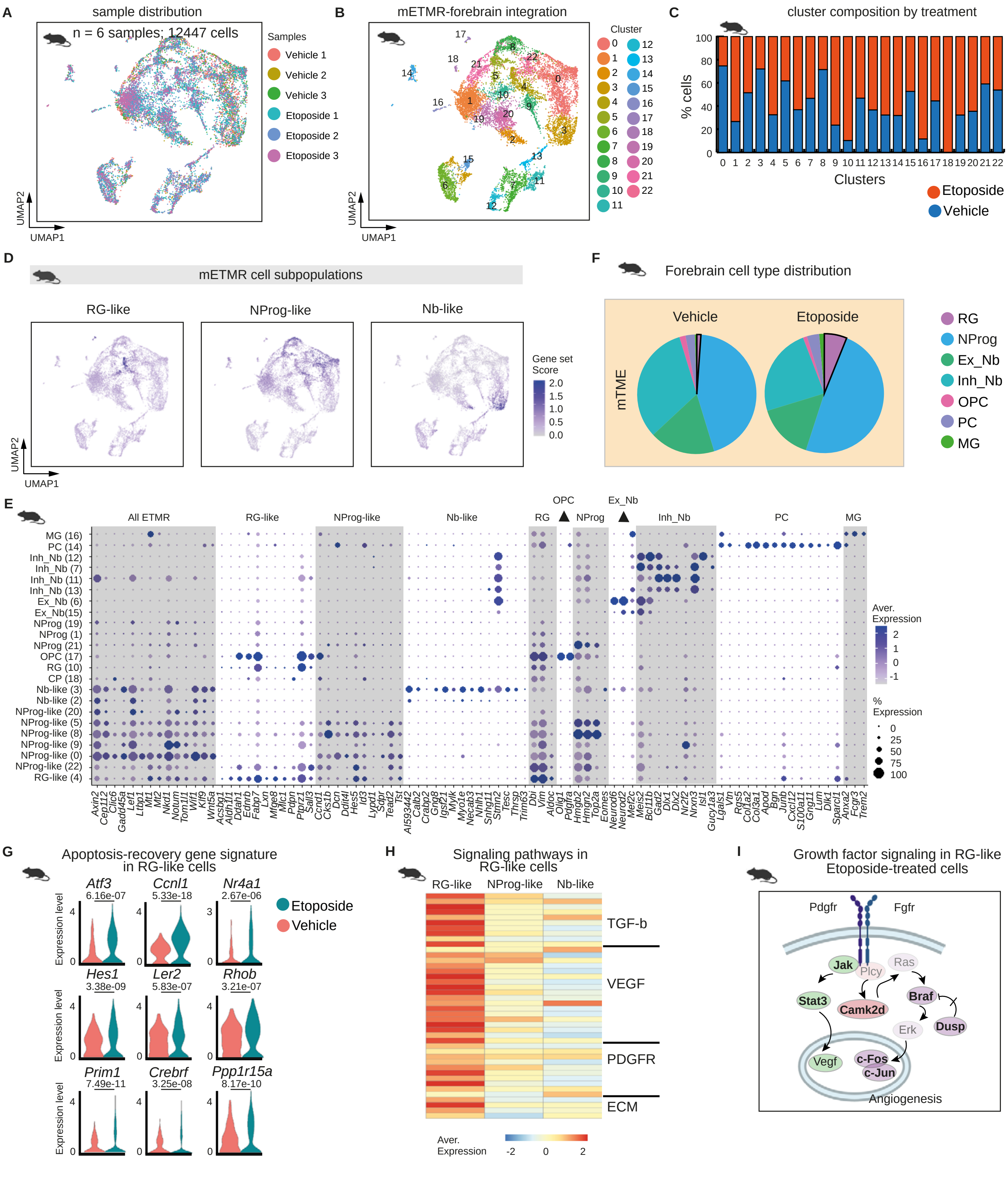

Supplementary Fig. 19. RG-like cells recover from etoposide-induced apoptosis upregulating PDGFR, TGFb, VEGF and ECM-related pathways in vivo. **A**, UMAP showing the distribution of the cells by sample. The integrated dataset is composed of mETMR-harboring embryo forebrains (mETMR-FB) either treated with Etoposide (n = 3) or with vehicle (n = 3). Color-coded by sample. **B**, UMAP representation of the 23 cellular states identified by unsupervised clustering and corres-pon-ding to cell types or subtypes within the dataset. Color-coded by original Seurat clustering. **C**, Bar graph showing the relati-ve distribution of the cells in the dataset by condition etoposide versus vehicle and color-coded by condition. Clusters are shown in more detail in (C). **D**, Panel depicting the gene signature enrichment score of the ETMR subpopulations in the dataset. **E**, Dot plot showing the marker genes defining the cell annotation. The corresponding cluster numbers are presented in parentheses. The size of the dots represents the percentage of cells within a cluster expressing the gene. The color scale shows the average expres-si-on level of the indicated gene per cell subtype. **F**, Pie charts comparing the relative proportion of the FB cell types in the etopo-si-de-treated versus vehicle-treated samples. **G**, Violin plots depicting “apoptosis-recovery” differentially expressed genes between etoposide versus vehicle-treated RG-like cells, calculated using two-sided MAST statistical test; adj-p values are displayed. **H**, Heatmap depicting the differentially expressed signaling pathways enriched in the etoposide-treated RG-like cells compared with the other ETMR subpopulations. Color-coded by avera-ge gene expression. **I**, Scheme of Pdgfr signaling in etopo-side treated RG-like cells, based on the differentially expressed genes related to Fig. 6K. Sample type symbols in **A-I** created with Biorender.com

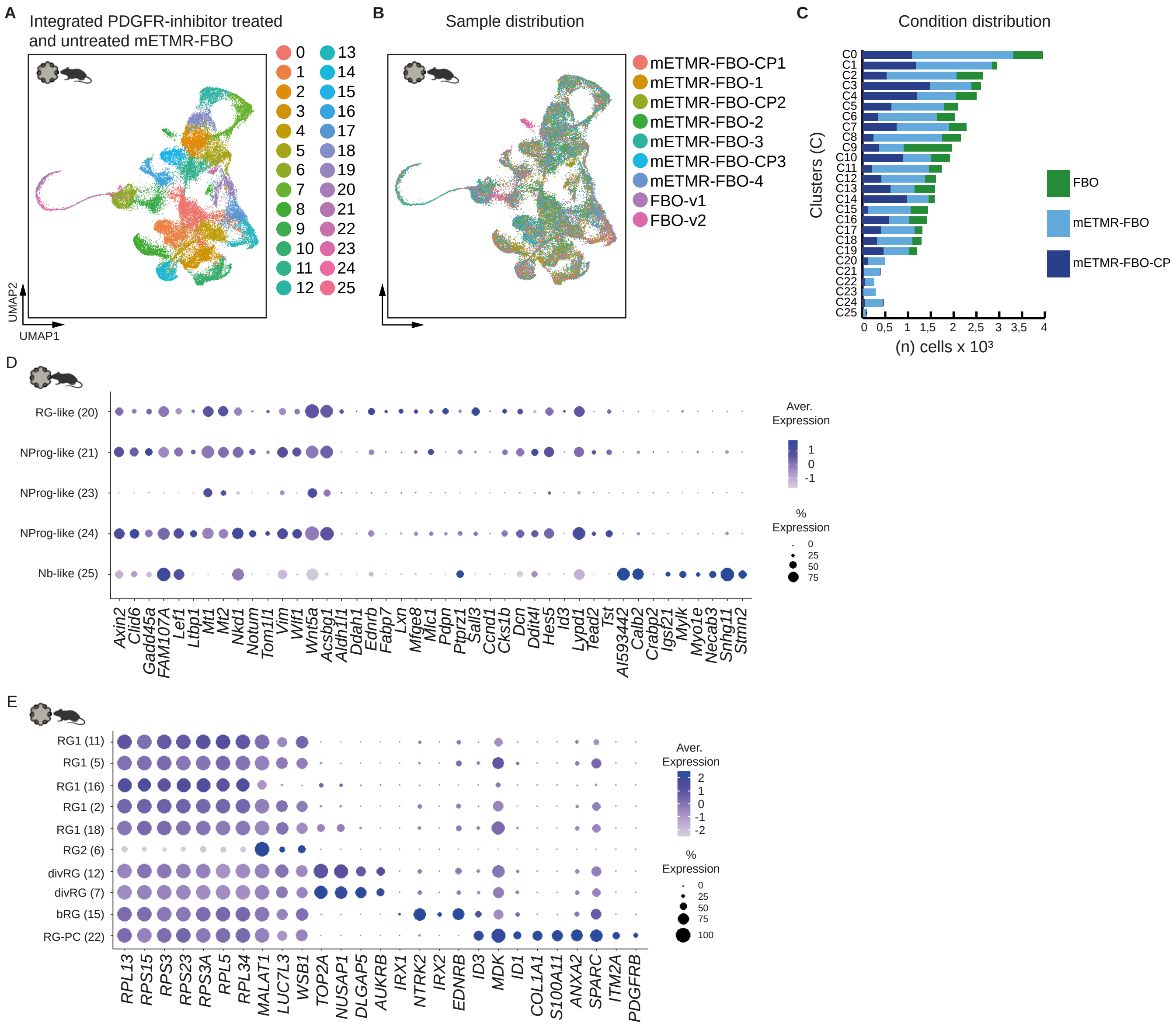

Supplementary Fig. 20. Cellular diversity in the scRNA-seq of mETMR-FBO PDGFR-inhibitor treatment. **A**, UMAP depicting the 26 distinct cellular states identified in the dataset by unsupervised clustering, representing cellular types or subtypes. Color-coded by cluster. **B**, UMAP presenting the integrated dataset cell distribution by sample: FBO (non-harboring ETMR, n = 2), mETMR-FBO DMSO-treated (n = 4) or mETMR-FBO-CP treated with the PDGFR-inhibitor CP673451 (n = 3). Color-coded by sample. **C**, Cellular distribution in dataset from (A-B) is balanced among the conditions analysed: i) FBO; ii) mETMR-FBO (DMSO control); iii) mETMR-FBO-CP. Color-coded by condition. **D**, Dot plot of the marker genes defining the ETMR cell subpopulations in the dataset. The corresponding cluster numbers are presented in parentheses (). The size of the dots represents the percentage of cells within a cluster expressing the gene. The color scale shows the average expression level of the indicated gene per cell subtype. **E**, Dot plot of representative marker genes defining the RG subpopulations found in the dataset. Sample type symbols in **A, B, D-E** created with Biorender.com

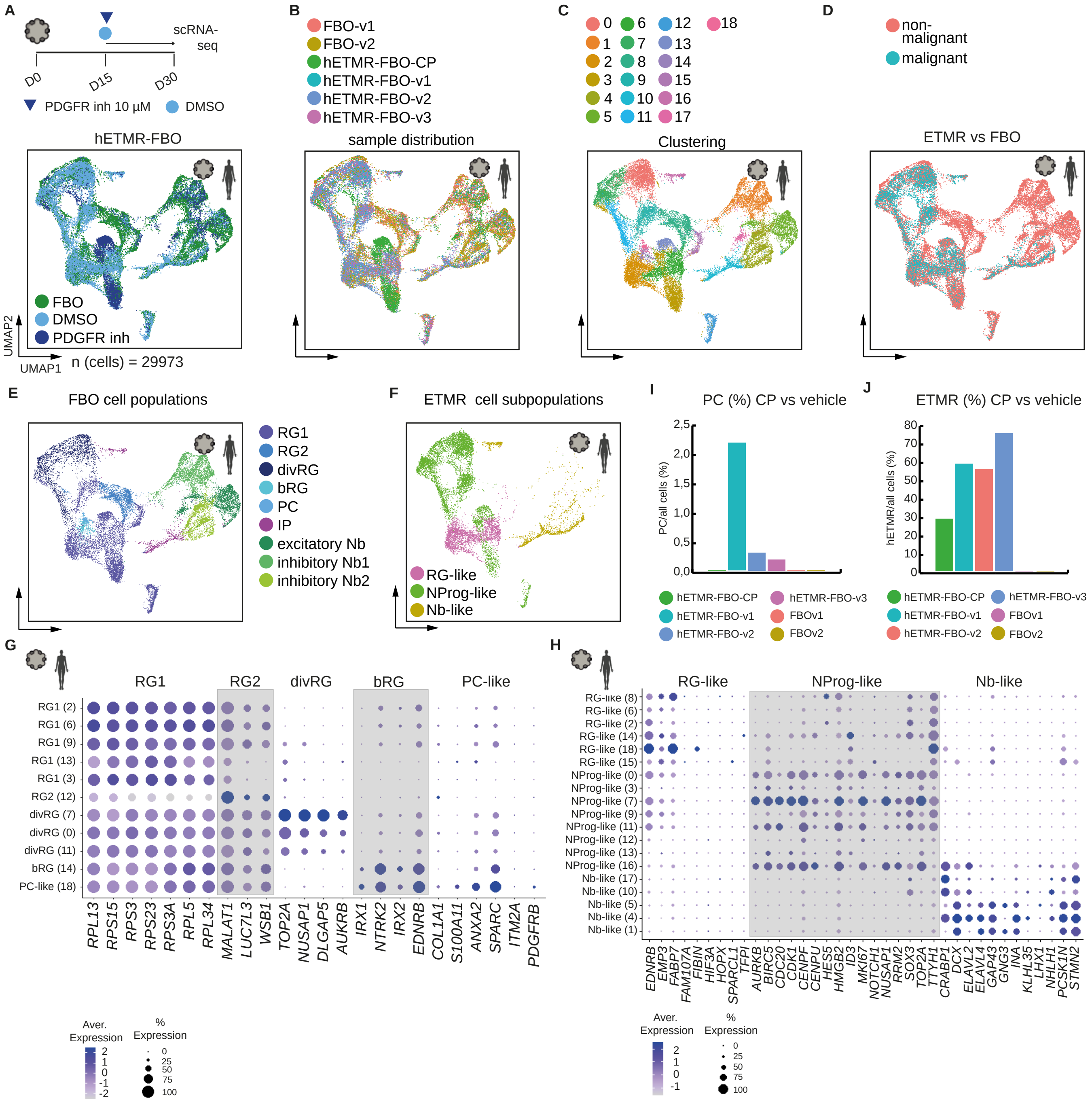

Supplementary Fig. 21. Cellular diversity in the scRNA-seq of hETMR-FBO PDGFR-inhibitor treatment. **A**, (upper panel) hETMR-FBOs were treated with PDGFR-inhibitor (PDGFR inh) CP-673451 10 µM or DMSO (vehicle) (from D15 to D30 and harvested for scRNA-seq. UMAP (lower panel) shows the 29,973 quality control-filtered cells in the dataset clustered by condition: hETMR-FBO PDGFR inh-treated or DMSO-treated and FBO-only (no tumor) untreated control. Color-coded by condition. **B**, UMAP presenting the integrated dataset cell distribution by sample: FBO (non-harboring ETMR, n = 2), hETMR-FBO-v (vehicle) (n = 3) or hETMR-FBO-CP treated with the PDGFR-inhibitor CP673451 (n = 1). Color-coded by sample. **C**, UMAP depicting the 19 distinct cellular states identified in the dataset by unsupervised clustering, representing cellu-lar types or subtypes. Color-coded by cluster. **D**, UMAP characterization of the malignant cells in the dataset, defined by the expression of Y-chromosome genes. Malignant cells in (green). Remaining non-malignant cells (pink) representing the FBO cellular population. **E**, Cell type annotation of the dataset. Color-coded by cell type. hETMR cells compose distinct clusters from the FBO. **F**, Clustering of the hETMR subpopulations. Color-coded by cell sub type. **G**, Dot plot of representative marker genes defining the RG subpopulations found in the dataset. The corresponding cluster numbers are presented in parentheses (). The size of the dots represents the percentage of cells within a cluster expressing the gene. The color scale shows the average expressi-on level of the indicated gene per cell subtype. **H**, Dot plot of the marker genes defining the hETMR cell subpopulations in the dataset. **I, J** Proportional enrichment of PC (I) or hETMR (J) cells to the total number of cells of each dataset, comparing the hETMR-FBO-CP treated sample with the three vehicle-treated samples. No PC gene expression was found in the PDG-FR-inh treated sample (I). Sample type symbols in **A-F, G, H** created with Biorender.com
