# Supplementary material for: ETMR stem-like state and chemo-resistance are supported by perivascular cells at single-cell resolution
Source: Nat Commun. 2025 Jun 25;16:5394. doi: 10.1038/s41467-025-60442-9 (PMC12198369; doi:10.1038/s41467-025-60442-9)
Supplement: Supplementary file 3 — Description of Additional Supplementary Files [file 41467_2025_60442_MOESM3_ESM.pdf]

Description of Additional Supplementary Files

Supplementary Data 1. Sample information and quality control for all normal and tumor scRNAseq samples. Sample/patient metadata, prepration protocols and sequencing quality parameters for initial data processing are reported.

Supplementary Data 2. Sample information and quality control for the spatial transcriptomics tissues. Sample/patient metadata, preparation protocol and sequencing quality parameters for initial data processing are reported.

Supplementary Data 3. Sample information and quality control for all normal and tumor FixedRNAseq samples. Sample/patient metadata, prepration protocols and sequencing quality parameters for initial data processing are reported.

Supplementary Data 4. Sample information for all tisssues used for IHC analysis. Sample/patient metadata is reported.

Supplementary Data 5. Cell-type specific canonical markers from the literature used to determine cluster identity.

Supplementary Data 6. Top 50 differentially expressed genes od the cell-types found in the sc- or sn-RNA-seq datasets of this study.

Supplementary Data 7. Gene signatures of ETMR and pericytes used as input for the celly type assignment in the scRNA-seq of the co-culture experiment and forebrain organoids.

Supplementary Data 8. Cluster information and quality control parameters for the sc/sn-RNA-seq datasets of this study.

Supplementary Data 9. Antibody information for the immunohistochemistry (IHC) and immunoflorescence (IF) analyses performed in this study.
