# Supplementary material for: ETMR stem-like state and chemo-resistance are supported by perivascular cells at single-cell resolution
Source: Nat Commun. 2025 Jun 25;16:5394. doi: 10.1038/s41467-025-60442-9 (PMC12198369; doi:10.1038/s41467-025-60442-9)
Supplement: Supplementary file 5 — Reporting Summary [file 41467_2025_60442_MOESM5_ESM.pdf]

nature portfolio

Corresponding author(s): Kornelius Kerl

Last updated by author(s): May 13, 2025

Reporting Summary

Nature Portfolio wishes to improve the reproducibility of the work that we publish. This form provides structure for consistency and transparency in reporting. For further information on Nature Portfolio policies, see our Editorial Policies and the Editorial Policy Checklist.

Statistics

For all statistical analyses, confirm that the following items are present in the figure legend, table legend, main text, or Methods section.

n/a	Confirmed	
☐	☒	The exact sample size (*n*) for each experimental group/condition, given as a discrete number and unit of measurement
☐	☒	A statement on whether measurements were taken from distinct samples or whether the same sample was measured repeatedly
☐	☒	The statistical test(s) used AND whether they are one- or two-sided<br>*Only common tests should be described solely by name; describe more complex techniques in the Methods section.*
☐	☒	A description of all covariates tested
☐	☒	A description of any assumptions or corrections, such as tests of normality and adjustment for multiple comparisons
☐	☒	A full description of the statistical parameters including central tendency (e.g. means) or other basic estimates (e.g. regression coefficient) AND variation (e.g. standard deviation) or associated estimates of uncertainty (e.g. confidence intervals)
☐	☒	For null hypothesis testing, the test statistic (e.g. F, t, r) with confidence intervals, effect sizes, degrees of freedom and P value noted<br>*Give P values as exact values whenever suitable.*
☒	☐	For Bayesian analysis, information on the choice of priors and Markov chain Monte Carlo settings
☒	☐	For hierarchical and complex designs, identification of the appropriate level for tests and full reporting of outcomes
☐	☒	Estimates of effect sizes (e.g. Cohen's d, Pearson's r), indicating how they were calculated

Our web collection on statistics for biologists contains articles on many of the points above.

Software and code

Policy information about availability of computer code

Data collection	1) single-cell RNA seq, FixedRNA seq and Visium spatial transcriptomics data were sequenced using Illumina NextSeq 2000 technology. The raw sequencing data was demultiplexed and aligned to either human or mouse reference genome and transformed into a cell (or spot)-feature matrix using Cell Ranger software from 10xGenomics.
Data analysis	Count data was analysed using default parameters with Seurat v4.0.5 and R v4.0.5. This study used NO custom code or software. All codes and softwares used in this study were previously established and are deposited in github and can be found in the following links: : 1) Seurat: https://satijalab.org/seurat/ or https://cloud.r-project.org/web/packages/Seurat/index.html; 2) CellPhoneDB V2: https://github.com/Teichlab/cellphonedb; 3) InterCellar: https://www.bioconductor.org/packages/release/bioc/html/InterCellar.html; 4) CellRanger: https://support.10xgenomics.com/single-cell-gene expression/software/pipelines/latest/installation; 5) CONICSmat: https://github.com/diazlab/CONICS.<br>2) Quantitative immunohistochemistry was performed using Vectra® 3.0 multispectral imaging system and the image analysis software Phenochart™ and InForm® (PerkinElmer) classifier, from Akoya Biosciences.<br>3) Whole-mount-staining imaging was processed and analysed in Columbus version 2.6.0 (Perkin Elmer) using a maximum projection of all confocal planes after automatic flatfield correction.<br>4) Multiplex immunofluorescense was performed using the MACSima Imaging System (Miltenyi) according to its user manual and image analysis was performed with the software MACS iQ View (Miltenyi Biotec).

For manuscripts utilizing custom algorithms or software that are central to the research but not yet described in published literature, software must be made available to editors and reviewers. We strongly encourage code deposition in a community repository (e.g. GitHub). See the Nature Portfolio guidelines for submitting code & software for further information.

Data

Policy information about availability of data

All manuscripts must include a data availability statement. This statement should provide the following information, where applicable:

- Accession codes, unique identifiers, or web links for publicly available datasets
- A description of any restrictions on data availability
- For clinical datasets or third party data, please ensure that the statement adheres to our policy

Raw single-cell RNA-seq and spatial transcriptomic data from this manuscript have been submitted to GEO with the accession number GSE224478 [https://www.ncbi.nlm.nih.gov/geo/query/acc.cgi?acc=GSE224478].
Raw FixedRNA-seq data is deposited under the accession number GSE254819 [https://www.ncbi.nlm.nih.gov/geo/query/acc.cgi?acc=GSE254819].
Single-cell RNA-seq data from one human ETMR sample were obtained from Jessa et al. (2019) and are available in the European Genome-Phenome Archive under accession number EGAS00001003368 [https://ega-archive.org/studies/EGAS00001003368]. Bulk-RNA sequencing data was obtained from Lambo et al. (2019) and is publicly available in GEO database under the accession number GSE122077 [https://www.ncbi.nlm.nih.gov/geo/query/acc.cgi?acc=GSE122077]. Murine cell lines can be readily available upon request to the corresponding author. ETMR-forebrain organoids can be obtained upon request to the corresponding author. Source data are provided with this paper.

Research involving human participants, their data, or biological material

Policy information about studies with human participants or human data. See also policy information about sex, gender (identity/presentation), and sexual orientation and race, ethnicity and racism.

Reporting on sex and gender	Sex / gender of human samples are provided in the Supplementary Data 1-4. All provided sex / gender information were self - reported. Neither sex or gender were considered in this study design, as we deal with a rare disease and all available information was used, independent on clinical, social or cultural parameters.
Reporting on race, ethnicity, or other socially relevant groupings	This study did not involve human participants, but used human biological material. No information regarding the social background or gender identity of the donors is available or provided.
Population characteristics	This study includes pediatric patients from 0 to 5 years old, both sexes (M / F), with the diagnosis of a brain tumor classified as Embryonal Tumor with Multilayered Rosettes (ETMR) by a reference pathology with standard clinical workup, 850k methylation classification, and molecular inversion probe CNV showing C19MC amplification.
Recruitment	Experiments were performed on previously available archived patient material. No patients were recruited specifically for this study.
Ethics oversight	Fresh tumor specimens were collected with written consent of the respective patients´ parents or adult legal representatives per protocols approved by the Ethics Committee Münster (2017-261-f-S). Formalin-fixed paraffin-embedded (FFPE) tumor specimens were provided by Prof. Ulrich Schüller (University Medical Center Hamburg-Eppendorf) and Prof. Dr. Jens Schittenhelm (University of Tübingen, Germany) with the written consent of the respective patients´ parents or adult legal representatives. As this study describes a very rare disease, we included all patients and did not select for age or sex/gender in advance. Sex/gender of patients was determined based on self-report.

Note that full information on the approval of the study protocol must also be provided in the manuscript.

Field-specific reporting

Please select the one below that is the best fit for your research. If you are not sure, read the appropriate sections before making your selection.

☒ Life sciences ☐ Behavioural & social sciences ☐ Ecological, evolutionary & environmental sciences

For a reference copy of the document with all sections, see nature.com/documents/nr-reporting-summary-flat.pdf

Life sciences study design

All studies must disclose on these points even when the disclosure is negative.

Sample size	For patient tumors, n = 19 (considering all the experiments); for murine tumors, n = 8 embryos; for experiments using cell lines = n 4; for experiments using 3D organoids, n = 33 (in total). Human sample size was determined by the available number of samples for study. The number of animals were defined as the minimum necessary to support our findings and limited by the number of animals authorized by animal authorities in the scope of this study. The number of cell lines and 3D organoids was defined by the number of conditions necessary to respond to our scientific questions. Whenever possible, we used >= 3 replicates per condition analysed.
Data exclusions	From all single-cell RNA sequencing datasets, we excluded from analysis all cells with low quality (< 200 genes / cell; > 25% mitocondrial genes), as described in the Methods section. No futher exclusions were used for this study.
Replication	We used 9 biological replicates for the human FixedRNAseq study and 2 other biological replicates for single-cell RNA-seq of human tumors, in

Replication	two independent cohorts. Among the murine single-cell cohorts, we used 4 biological replicates for E16.5 murine cohort and 3 biological replicates for a validation murine cohort at E18.5. We provide 3 biological replicates for ETMR spatial transcriptomics for analysis. Although the tissues are heterogeneous, integrated analysis successfuly support our main findings; 2 biological replicates for the each mono- and co-culture experiments; 2-3 biological replicates for mETMR-FBO dataset/condition analysed; 1-2 technical replicates for hETMR-FBO dataset / condition analysed (there is only one human ETMR cell line available in the scientific community); 3 biological replicates per condition for in vivo chemotherapy study; 1 biological replicate for multiplex immunofluorescene experiment. We succesfully replicated all the experiments for which replication was performed within this study.
Randomization	Our in vivo interWe didventional studies required comparisons between chemotherapy-treated and control animals. To reduce the size of our cohort to as small as possible, though keeping balanced numbers for comparisons (as per the "3R" animal research rules - "Reduce") we did not apply randomization.
Blinding	Our study required accurate identification of malignant and non-malignant samples; therefore, blinding was not possible for the analyses performed. For in vivo therapy experiments, both tumor-bearing mice and controls were treated in a blinded manner, as treatments were administered during the intra-uterine phase. However, due to the reasons outlined above, data analysis was not conducted in a blinded fashion.

Reporting for specific materials, systems and methods

We require information from authors about some types of materials, experimental systems and methods used in many studies. Here, indicate whether each material, system or method listed is relevant to your study. If you are not sure if a list item applies to your research, read the appropriate section before selecting a response.

Materials & experimental systems

n/a	Involved in the study
☐	☒ Antibodies
☐	☒ Eukaryotic cell lines
☒	☐ Palaeontology and archaeology
☐	☒ Animals and other organisms
☒	☐ Clinical data
☒	☐ Dual use research of concern
☒	☐ Plants

Methods

n/a	Involved in the study
☒	☐ ChIP-seq
☒	☐ Flow cytometry
☒	☐ MRI-based neuroimaging

Antibodies

Antibodies used

All antibodies used in the study are reported in the Supplementary Data 9, as follows:
Antibody Cat Nr. Manufacturer Dilution RRID Use
Ki67 ab15580 abcam 1:100 AB_443209 IHC
Nestin (murine) ab221660 abcam 1:2000 AB_2909415 IHC
Nestin (human) MAB5326 Millipore 1:3000 AB_2251134 IHC
Synaptophysin M7315 DAKO 1:500 - IHC
MAP2c M4403 Sigma 1:3000 AB_477193 IHC
SOX2 ab92494 abcam 1:200 AB_10585428 IHC
GFP ab290 abcam 1:500 AB_303395 IHC
NeuN MAB377 Chemicon 1:50 - IHC
LIN28A #3978 Cell Signaling 1:50 - IHC
Cre 908001 biolegend 1:100 AB_2565079 WB
beta-Catenin M3539 DAKO 1:100 - IHC
GFP ab13970 abcam 1:500 AB_300798 IF
MAP2 ab32454 abcam 1:500 AB_776174 IHC
Alexa Fluor 647 goat anti chicken # A-21449 ThermoFisher 1:1000 AB_2535866 IHC
Alexa Fluor 647 donkey anti rabbit # A-31573 ThermoFisher 1:1000 AB_2536183 IHC
CD13 38C12 Leica Biosystems 1:80 AB_563491 IHC
Ki_67 130-120-557 Miltenyi Biotec 1:100 AB_2784392 IF
Actin 130-123-363 Miltenyi Biotec 1:100 AB_2857593 IF
CD31 130-128-769 Miltenyi Biotec 1:100 AB_2904938 IF
SOX2 130-120-721 Miltenyi Biotec 1:100 AB_2784458 IF
MAP2 130-128-142 Miltenyi Biotec 1:100 AB_2905326 IF
Nestin ab187846 Abcam 1:100 - IF
PDGFRb ab32570 Abcam 1:100 AB_777165 IF

Validation

All antibodies listed above have been validated by the manufactures. The link for the manufacturer website is provided in the Supplementary Data 9 for every antibody listed above.

Eukaryotic cell lines

Policy information about cell lines and Sex and Gender in Research

Cell line source(s)	murine hGFAP-cre::Ctnnb1(ex3)Fl/+SmoM2Fl/+ derived cell lines were generated in house (ETMRT3, ETMRT4) and provided by Prof. Dr. U. Schüller (ETMR1, ETMRJ) at the University Medical Center Hamburg-Eppendorf – UKE, Germany. Those cell lines were generated from animal embryos, for which sex information is not available. The human cell line (BT-183) originally comes from a male patient and it was provided by Dr. Jennifer Chan at the University of Calgary, Calgary, Canada. Human induced pluripotent stem cells (hiPSC) originally come from a female donor, were characterized in a previous study (Reinhardt et al., 2013) and provided by Dr. Jan M. Bruder (Max Planck Institute for molecular Biomedicine, Münster, Germany).
Authentication	Cell lines were not authenticated
Mycoplasma contamination	Cell lines were negative for Mycoplasma
Commonly misidentified lines (See ICLAC register)	No commonly misidentified cell lines were used

Animals and other research organisms

Policy information about studies involving animals; ARRIVE guidelines recommended for reporting animal research, and Sex and Gender in Research

Laboratory animals	Genetically engineered mouse models (mus musculus) hGFAP-cre, SmoM2fl/fl, Ctnnb1(ex3)fl/fl, and a hGFAP-cre::Ctnnb1(ex3)fl/+SmoM2fl/+ strains with a yFP reporter gene under the control of SmoM2 promoter have been previously generated and described (Mao, 2001; Zhuo, 2006; Neumann, 2017). All animals were housed and bred at the Central Animal Experimentation Facility of the University of Münster (Münster, Germany), in accordance with institutional and governmental regulations. Crossing SmoM2fl/fl with Ctnnb1(ex3)fl/fl mice, we obtained the heterozygous SmoM2fl/+Ctnnb1(ex3) fl/+ strain, which harbors no phenotype. By crossing hGFAP-cre mice with SmoM2fl/flCtnnb1(ex3) fl/fl mice, we obtained hGFAP-cre::Ctnnb1(ex3) fl/+SmoM2 fl/+ mice, in which Wnt and Shh pathways were constitutively activated, leading to ETMR development. we obtained the forebrain of mice at either embryonal age of E16.5 or E18.5. Forebrains of mice at E16.5 were used for scRNA-seq, and those at E18.5 as a validation cohort for scRNA-seq and analysis of chemotherapy treatment. Information about every animal used in every experiment is provided in the Supplementary Data 1, by experiment type - Single-cell RNA-seq (Suppl. Data 1) and IHC (Suppl. Data 4).
Wild animals	No wild animals were used.
Reporting on sex	Mice at embryonal age was used and sex was not evaluated
Field-collected samples	No field-collected samples were used.
Ethics oversight	This study has received approaval from animal authorities - LANUV, Government of NRW, Germany. Reference numbers 81-02.04.2018.A214; 81-02.04.2020.A474; 81-02.04.2021.A258

Note that full information on the approval of the study protocol must also be provided in the manuscript.

Plants

Seed stocks	No plants were used in this study
Novel plant genotypes	See above
Authentication	See above
